# Efficacy and safety of Shenxiong-Xinmaikang Decoction in patients with stable angina pectoris: a real-world prospective observational study

**DOI:** 10.3389/fphar.2025.1591959

**Published:** 2025-07-30

**Authors:** Yanchen Dong, Kaojuan Zeng, Yongzhen Liang, Guoping Xie, Donghui Liang

**Affiliations:** ^1^ Department of Traditional Chinese Medicine, The Third Affiliated Hospital, Southern Medical University, Guangzhou, China; ^2^ Department of Traditional Chinese Medicine, Zhujiang Hospital, Southern Medical University, Guangzhou, China; ^3^ School of Traditional Chinese Medicine, Southern Medical University, Guangzhou, China; ^4^ Department of Respiratory Medicine, Huizhou Central People’s Hospital, Huizhou, China

**Keywords:** Shenxiong-Xinmaikang Decoction, stable angina pectoris, traditional Chinese medicine, inverse probability treatment weighting, efficacy

## Abstract

**Background:**

Shenxiong-Xinmaikang Decoction (SXXMKD), a modified traditional Chinese herbal formula, is widely used in clinical practice for stable angina pectoris (SAP). However, robust clinical evidence supporting its efficacy and safety is lacking. This study aimed to evaluate the real-world effectiveness and safety of SXXMKD as an adjunct to standard therapy for SAP.

**Methods:**

This was a prospective, single-center, real-world observational study conducted between 2019 and 2023, enrolling 219 SAP patients (Registry: ChiCTR2100052872). Patients received either standard care alone (standard biomedical treatment group, SBMT group; *n* = 101) or SXXMKD combined with standard care (combined Traditional Chinese Medicine, CTCM group; *n* = 118). To minimize selection bias and balance baseline covariates, we applied inverse probability of treatment weighting (IPTW). Clinical outcomes were assessed at short- and long-term follow-up.

**Results:**

After IPTW adjustment, baseline characteristics were well-balanced between the groups. Compared to the SBMT group, the CTCM group demonstrated significant improvements in angina symptom scores, electrocardiogram readings, and left ventricular ejection fraction (LVEF). Furthermore, the CTCM group showed lower levels of low-density lipoprotein cholesterol (LDL-C), creatine kinase (CK), high-sensitivity C-reactive protein (hs-CRP), and cardiac troponin I (cTnI). While the incidence of cardiovascular events was reduced in the CTCM group, the difference in cardiovascular event-free survival did not reach statistical significance.

**Conclusion:**

In a real-world setting, the addition of SXXMKD to standard biomedical treatment for SAP was safe and associated with improved symptomatic, functional, and biochemical outcomes. These findings suggest that SXXMKD is a promising adjunctive treatment for improving clinical outcomes in patients with stable angina.

**Clinical trial registration:**

https://www.chictr.org.cn/, identifier: ChiCTR2100052872.

## 1 Introduction

Stable angina pectoris (SAP) is a clinical syndrome of transient myocardial ischemia, typically presenting as episodic chest pain or tightness. The presence of angina is a well-established predictor of major adverse cardiovascular events, including myocardial infarction and mortality (Joshi and de Lemos, 2021; Valgimigli and Biscaglia, 2014). Therefore, the primary goals of SAP management are to alleviate symptoms and, critically, to prevent disease progression and reduce the risk of future cardiovascular events. Standard pharmacological interventions include nitrates, antiplatelet agents, beta-blockers, and lipid-lowering drugs, which form the cornerstone of current therapy (Joshi and de Lemos, 2021).

However, standard biomedical treatments are not without limitations. Their long-term use can be associated with adverse effects, such as drug-induced liver injury or an increased risk of bleeding, which may compromise patient adherence (Walker et al., 2020; Wang et al., 2022). This underscores the ongoing need for novel therapeutic strategies that can offer additional benefits or an improved safety profile. Traditional Chinese Medicine (TCM) offers a complementary approach, with studies indicating that integrative treatment can enhance symptomatic relief, reduce post-intervention restenosis, and minimize adverse events in patients with angina (Li et al., 2022a).

One such TCM formulation is the Shenxiong-Xinmaikang Decoction (SXXMKD), a well-established multi-herb formula used for the treatment of SAP. Our previous work, using network pharmacology and molecular docking, provided a preliminary mechanistic rationale for its use, suggesting that SXXMKD’s key components may act primarily through anti-inflammatory and lipid-regulating pathways (Dong et al., 2024). This pre-clinical evidence offers a strong basis for its clinical investigation.

Despite this promising mechanistic groundwork, robust clinical evidence for the efficacy and safety of SXXMKD remains scarce. A significant gap exists between its traditional use, pre-clinical plausibility, and the evidence-based validation required for modern clinical practice. Therefore, this real-world observational study was designed to evaluate the effectiveness and safety of SXXMKD as an adjunctive therapy for patients with SAP.

## 2 Materials and methods

### 2.1 Study design

This study represents a single-center, prospective cohort analysis conducted at Zhujiang Hospital of Southern Medical University. Ethical approval for this study was obtained from the Ethics Committee of Zhujiang Hospital, Southern Medical University (2022-KY-013-01), and the trial was registered with the *Chinese Clinical Trial Registry* (ChiCTR2100052872). Before their participants in the study, all patients provided written informed consent. Subjects were recruited from the Departments of Cardiology and TCM to identify patients who met the pre-defined inclusion criteria. Patients were divided into the combined Traditional Chinese medicine (CTCM) group and the standard biomedical treatment group (SBMT) group according to whether they used SXXMKD or not.

### 2.2 Setting and participants

The inclusion criteria for this study were as follows: (1) Patients aged between 18 and 80. (2) Following the “Guidelines for Primary Diagnosis and Treatment of Stable Coronary Heart Disease (2020)” (Association and House, 2021), the “2013 ESC Guidelines for the Management of Stable Coronary Heart Disease” (Task Force et al., 2013), the “2012 ACC/AHA/ACP/AATS/PCNA/SCAI/STS Guidelines for the Diagnosis and Treatment of Patients with Stable Ischemic Heart Disease” (Levine et al., 2016), and the “Path of Noninvasive Imaging Experts Consensus on the Stability of Coronary Heart Disease (2017)” (Cardiovascular Imaging Group SoC et al., 2017), the clinical presentation of SAP within the context of coronary heart disease (CHD) is characterized as follows: Angina typically affects the chest, especially behind the breastbone or on the left side, but can also be felt in the upper stomach, throat, or neck and sometimes spreads to the left arm. The pain is typically described as a pressing, choking, burning, or heavy sensation, rather than a sharp, stabbing pain. It comes and goes in a few minutes, is often triggered by activity or stress, and usually goes away quickly with rest or a nitroglycerin tablet under the tongue. Among the following conditions: (1) Chest pain is accompanied by clear ST-T changes on the Electrocardiogram (ECG), either as S segment depression or T wave inversion at rest, with “false normalization” during pain. Alternatively, a 24-h Holter ECG may show ST-T alterations matching the symptoms. (2) Coronary CT or angiography reveals that at least one major coronary artery or its main branch has a stenosis exceeding 50%. (3) An abnormal resting electrocardiogram, characterized by conditions such as left bundle branch block (LBBB), an ST segment depression exceeding 1 mm, paced rhythm, or pre-excitation syndrome, among others. (4) For those with inconclusive ECGs, a positive stress test is used; the Bruce protocol is applied, and a positive test result is required. (5) Patients with a stable disease trajectory following ischemic cardiomyopathy or acute coronary syndrome, with at least 6 months elapsed since the initial event. (3) Can provide contact information to cooperate with clinical follow-up.

The exclusion criteria for this study were as follows: (1) Incomplete inpatient or outpatient medical records; (2) Chest pain attributable to non-cardiac conditions such as myocarditis, cardiac neurosis, gastroesophageal reflux disease, cervical spondylosis, hyperthyroidism, intercostal neuralgia, rheumatic fever, syphilis, congenital coronary anomalies, or hypertrophic cardiomyopathy, aortic stenosis, or insufficiency; (3) Concurrent severe comorbidities, including but not limited to severe arrhythmias, advanced heart failure (NYHA Class III-IV), severe pulmonary insufficiency (PaO_2_ <60 mmHg), moderate to severe hepatic insufficiency (aminotransferase levels three times the upper limit of normal), moderate to severe renal insufficiency, acute cerebrovascular events, or severe primary endocrine or hematopoietic disorders; (4) History of psychiatric illness, dementia, alcoholism, or substance dependence; (5) Treatment with SXXMKD-based decoctions for less than 2 weeks or concurrent use of other TCM compounds or therapies such as acupuncture and massage during the study period.

### 2.3 Interventions

The SBMT group adheres to a standard protocol for secondary prevention of CHD. This regimen includes: (1) Antiplatelet medications, such as aspirin enteric-coated tablets and clopidogrel bisulfate tablets, to prevent blood clots. (2) Lipid-lowering drugs, including atorvastatin calcium tablets and rosuvastatin calcium tablets, to manage cholesterol levels. (3) ACE inhibitors, like enalapril maleate tablets, to lower blood pressure and reduce the workload on the heart. Angiotensin II receptor antagonists, such as irbesartan tablets, also help manage blood pressure. (4) Beta blockers, exemplified by metoprolol succinate sustained-release tablets, to control heart rate and blood pressure. (5) Long-acting nitrates, such as isosorbide mononitrate, to alleviate chest pain and improve blood flow. (6) Anti-myocardial ischemia drugs, including ivabradine and trimetazidine, reduce the heart’s oxygen demand and improve its function.

The CTCM group added SXXMKD to the standard biomedical treatment protocol. SXXMKD is a polyherbal formulation composed of *Salvia miltiorrhiza Bunge [Lamiaceae]* (Dan Shen in Chinese), *Astragalus mongholicus Bunge [Fabaceae]* (Huang Qi in Chinese), *Pueraria montana* var. *lobata (Willd.) Maesen & S.M.Almeida ex Sanjappa & Predeep [Fabaceae]* (Ge Gen in Chinese), *Panax notoginseng (Burkill) F.H.Chen [Araliaceae]* (San Qi in Chinese), *Ligusticum chuanxiong Hort. [Apiaceae]* (Chuan Xiong in Chinese), *Pseudostellaria heterophylla (Miq.) Pax ex Pax et Hoffm. [Caryophyllaceae]* (Tai Zi Shen in Chinese), *Dolomiaea costus (Falc.) Kasana & A.K.Pandey [Asteraceae]* (Mu Xiang in Chinese), *Crataegus pinnatifida Bunge [Rosaceae]* (Shan Zha in Chinese), and *Rhodiola rosea L. [Crassulaceae]* (Hong Jing Tian in Chinese) (Table 1). The decoction was prepared by boiling the herbs in water, with Mu Xiang added during the final stages to preserve its volatile components. Patients are advised to take the medication twice daily, in the morning and evening, to maintain a consistent therapeutic effect. The TCM treatment is further individualized based on the specific symptoms presented by the patient.

**TABLE 1:** Botanical composition and source information of Shenxiong-Xinmaikang Decoction (SXXMKD).

Full botanical drug species name	Chinese botanical drugs name	Origin	Harvesting time	WFO number	Part of botanical drugs	Processing method	Drying method
*Salvia miltiorrhiza Bunge [Lamiaceae]*	Dan Shen	Sichuan	November	0000301608	Root and rhizome	Cutting	Sun-drying/Oven-drying
*Astragalus mongholicus Bunge [Fabaceae]*	Huang Qi	Gansu	November	0000185834	Root	Cutting	Sun-drying/Oven-drying
*Pueraria montana* var. *lobata (Willd.) Maesen & S.M.Almeida ex Sanjappa & Predeep [Fabaceae]*	Ge Gen	Guangdong	July	0000182939	Root	Cleaning and Sorting	Sun-drying/Oven-drying
*Panax notoginseng (Burkill) F.H.Chen [Araliaceae]*	San Qi	Yunnan	July	0000529277	Root and rhizome	Cutting	Sun-drying/Oven-drying
*Ligusticum chuanxiong Hort. [Apiaceae]*	Chuan Xiong	Sichuan	June	0000362358	Rhizome	Cutting	Sun-drying/Oven-drying
*Pseudostellaria heterophylla (Miq.) Pax ex Pax et Hoffm. [Caryophyllaceae]*	Tai Zi Shen	Fujian	February	0000395657	Tuberoid Root	Cleaning and Sorting	Sun-drying/Oven-drying
*Dolomiaea costus (Falc.) Kasana & A.K.Pandey [Asteraceae]*	Mu Xiang	Yunnan	November	0000521856	Root	Cutting	Sun-drying/Oven-drying
*Crataegus pinnatifida Bunge [Rosaceae]*	Shan Zha	Shandong	November	0000988959	Fruit	Cleaning and Sorting	Sun-drying/Oven-drying
*Rhodiola rosea L. [Crassulaceae]*	Hong Jing Tian	Xizang	September	0000399342	Root and rhizome	Cutting	Sun-drying/Oven-drying

For instance, Tai Zi Shen and Radix Codonopsis Pilosulae are utilized based on the unique requirements of different conditions. Patients experiencing poor sleep quality may have their regimen augmented with Semen Ziziphi Spinosae and Prunus mume Siebold & Zucc. For those presenting with lumbago pain, Eucommia ulmoides Oliver and Achyranthes bidentata Blume are added to alleviate discomfort. Lastly, for patients with loose stools, Citrus reticulata Blanco and Dioscorea opposita Thunb are incorporated to regulate intestinal function. Medication is to be taken continuously for at least 2 weeks.

### 2.4 Plant material sourcing, authentication, and quality control

A rigorous, multi-layered quality control system was prospectively designed and implemented to ensure the identity, quality, and batch-to-batch consistency of the Shenxiong-Xinmaikang Decoction (SXXMKD) used in this study. All nine raw herbal materials were procured through the official supply chain of Zhujiang Hospital, Southern Medical University, from Guangdong Yuan Sheng Tai Pharmaceutical Co., Ltd. Upon receipt, each batch was sampled and subjected to a comprehensive authentication process by qualified pharmacists at the hospital’s Traditional Chinese Medicine (TCM) Pharmacy, which is certified for Good Clinical Practice (GCP).

The authentication protocol for each herb was conducted in accordance with the Chinese Pharmacopoeia (2020 edition) and included three key stages: (1) Macroscopic and microscopic examination to confirm botanical identity; (2) A qualitative Thin-Layer Chromatography (TLC) test against a reference standard to verify the species and rule out common adulterants; and (3) A quantitative High-Performance Liquid Chromatography (HPLC) assay to determine the content of a major bioactive marker compound, ensuring potency and chemical consistency. This systematic approach was applied to all nine herbs, and only batches that passed all quality specifications were approved. Voucher specimens from each authenticated batch were cataloged and deposited at the herbarium of the TCM Pharmacy for reference.

For phytochemical analysis, a parallel batch of the decoction was lyophilized (freeze-dried) to obtain a fine, homogenous powder. This analytical work, including the generation of liquid chromatography-tandem mass spectrometry (LC-MS/MS) analysis and UV-Vis fingerprints (see Supplementary Figures S1, S2), was conducted to identify the components of SXXMKD and comprehensively characterized the composition of the complex chemical components, including their relative contents in the compound formula (Heinrich et al., 2022). This provided a comprehensive chemical characterization of the formula administered to patients.

### 2.5 Outcomes measures

#### 2.5.1 Baseline data

Baseline data encompass the following categories: (1) Demographic information: This includes patient identifiers such as name, identification number, age, gender, and contact details. (2) Cardiovascular disease history: This comprises details on prior revascularization surgeries, episodes of heart failure, and a history of hypertension. (3) Cardiovascular risk factors: Assessments of body mass index, smoking history, family history of cardiovascular disease, diabetes, and dyslipidemia are recorded. (4) History of other comorbidities: Information on chronic obstructive pulmonary disease (COPD), cerebral infarction, liver disease, renal failure, anxiety, and depression is documented. (5) Medication history: A record of prior medication use, including antiplatelet drugs, nitrates, beta-blockers, statins, angiotensin-converting enzyme inhibitors, and proton pump inhibitors, is maintained.

#### 2.5.2 Primary outcome measures

The primary endpoint was the overall improvement rate of angina pectoris symptoms. Symptomatology at baseline and post-treatment was documented using the Angina Symptom Rating Scale (Supplementary Table S1). Following the calculation of scores, the differences in angina symptom scores between the two groups were compared based on the efficacy evaluation criteria outlined in Supplementary Table S2, ultimately determining the total effective rate of angina symptom improvement.

#### 2.5.3 Secondary outcome measure

ECG: Assessments were conducted following the “Angina Pectoris of Coronary Heart Disease and Evaluation Criteria of Electrocardiogram Efficacy” (Supplementary Table S3). Blood Lipid Profiles: These were evaluated based on the “Guiding Principles for Clinical Research of New Chinese Medicine published in 2002” (Supplementary Table S4). Other Laboratory Indicators: Additional parameters included left ventricular ejection fraction (LVEF), glycated hemoglobin, N-terminal B-type natriuretic peptide precursor (NT-pro-BNP), and high-sensitivity C-reactive protein (hs-CRP). Post-Treatment Follow-Up: Patients were monitored for 2–56 months post-medication through various means, including outpatient clinics, telephone, and WeChat. During this period, the frequency, severity, and duration of angina pectoris attacks, readmission or revisit rates, cardiovascular events (such as angina pectoris, myocardial infarction, and revascularization), and patient mortality were meticulously documented.

### 2.6 Safety and adverse event monitoring

#### 2.6.1 Adverse reaction

According to the WHO Adverse Reaction Terminology, the case records document whether patients experience any adverse reactions: Grade 1: Safe, with no adverse reactions. Grade 2: Relatively safe, with mild laboratory abnormalities, allergic reactions, headaches, dizziness, somnolence, gastrointestinal symptoms, etc., and no need for any treatment to continue therapy. Grade 3: Significant laboratory abnormalities, moderate allergic reactions, headaches, dizziness, somnolence, gastrointestinal symptoms, etc., and if symptoms are relieved after symptomatic treatment, continue therapy. Grade 4: Severe allergic reactions or other serious medical events that can lead to disability, malformation, cancer, hospitalization, or even life-threatening conditions.

#### 2.6.2 Safety evaluation indicators

Indicators include albumin, alanine aminotransferase, hemoglobin, platelet count, prothrombin time, and creatinine, which are measured before and after treatment (The incidence rate of adverse reactions = The number of adverse reaction cases/The total number of instances * 100%).

### 2.7 Sample size calculation

The sample size was estimated using a hypothesis-testing framework for comparing two independent proportions. The primary endpoint was the overall improvement rate of angina pectoris symptoms. Based on prior literature and retrospective data from our center, the effective rate was estimated to be 43.19% for standard biomedical treatment alone and 62.3% for the combined therapy with TCM. Using a two-sided test with *α* = 0.05 and power (1–β) = 0.80, a sample size of 104 per group (total *n* = 208) was calculated via the two-proportion chi-square test in PASS 15.0 (NCSS, LLC). No dropout buffer was applied; instead, a real-time dynamic sample size management strategy was adopted. The final sample size was determined by the number of patients who completed full follow-up to ensure the validity of the results.

### 2.8 Statistical analysis

The normality of the measurement data was assessed using the Kolmogorov-Smirnov test. For variables that were normally distributed, comparisons were made using the two-tailed t-test, with results expressed as mean ± standard deviation (SD). Non-normally distributed variables were analyzed with the Wilcoxon rank-sum test, and results were presented as median (interquartile range). The data analysis comparing the results before and after treatment in each group was performed using a paired two-sample t-test. Categorical data comparisons were performed using the Chi-square test or, when appropriate, the Fisher exact test.

To mitigate the potential selection bias and ensure equivalence in baseline characteristics between the two patient cohorts, we employed inverse probability of treatment weighting (IPTW). The propensity model encompassed a range of variables, including age, sex, history of other cardiovascular diseases, hyperlipidemia, co-existing anxiety or depression, smoking history, body mass index (BMI), comorbidity, LVEF, glycosylated hemoglobin (HbA1c), NT-pro-BNP, hs-CRP, alanine aminotransferase (ALT), albumin (ALB), hemoglobin (Hb), platelet count (PLT), prothrombin time (PT), creatinine, and angina symptom score. Propensity scores were derived from a multiple logistic regression analysis, and the IPTW method constructed a virtual cohort by assigning weights based on the inverse probability of treatment, as determined by these propensity scores. Compared to propensity score matching (PSM), IPTW preserves the original sample size, eliminating the need to exclude any participants. The balance of baseline characteristics was assessed using the standardized mean difference (SMD), with an SMD ≤0.1 indicating an optimal balance.

The Kaplan-Meier method was utilized to estimate cardiovascular event (CVE)-free survival, with the log-rank test employed for comparative analysis. Statistical significance was defined as a P-value less than 0.05. Data analysis was conducted using RStudio for Windows, version 4.0.5 (RStudio Inc., Boston, MA, United States), and SPSS Statistics, version 25.0 (IBM Corp., Armonk, NY, United States).

## 3 Results

### 3.1 Participants characteristics

A total of 583 patients diagnosed with SAP were admitted to Zhujiang Hospital of Southern Medical University between June 2019 and December 2023. After excluding cases with incomplete data (164 cases), those who took TCM for less than 2 weeks (87 cases), and patients who met other exclusion criteria (113 cases), a total of 219 patients who fulfilled the inclusion criteria were enrolled in this study. Within this cohort, 118 patients received SXXMKD in conjunction with standard biomedical treatment in the Department of TCM (CTCM group), while 101 patients were treated with standard biomedical therapy in the Cardiology Department (SBMT group).

**FIGURE 1:**
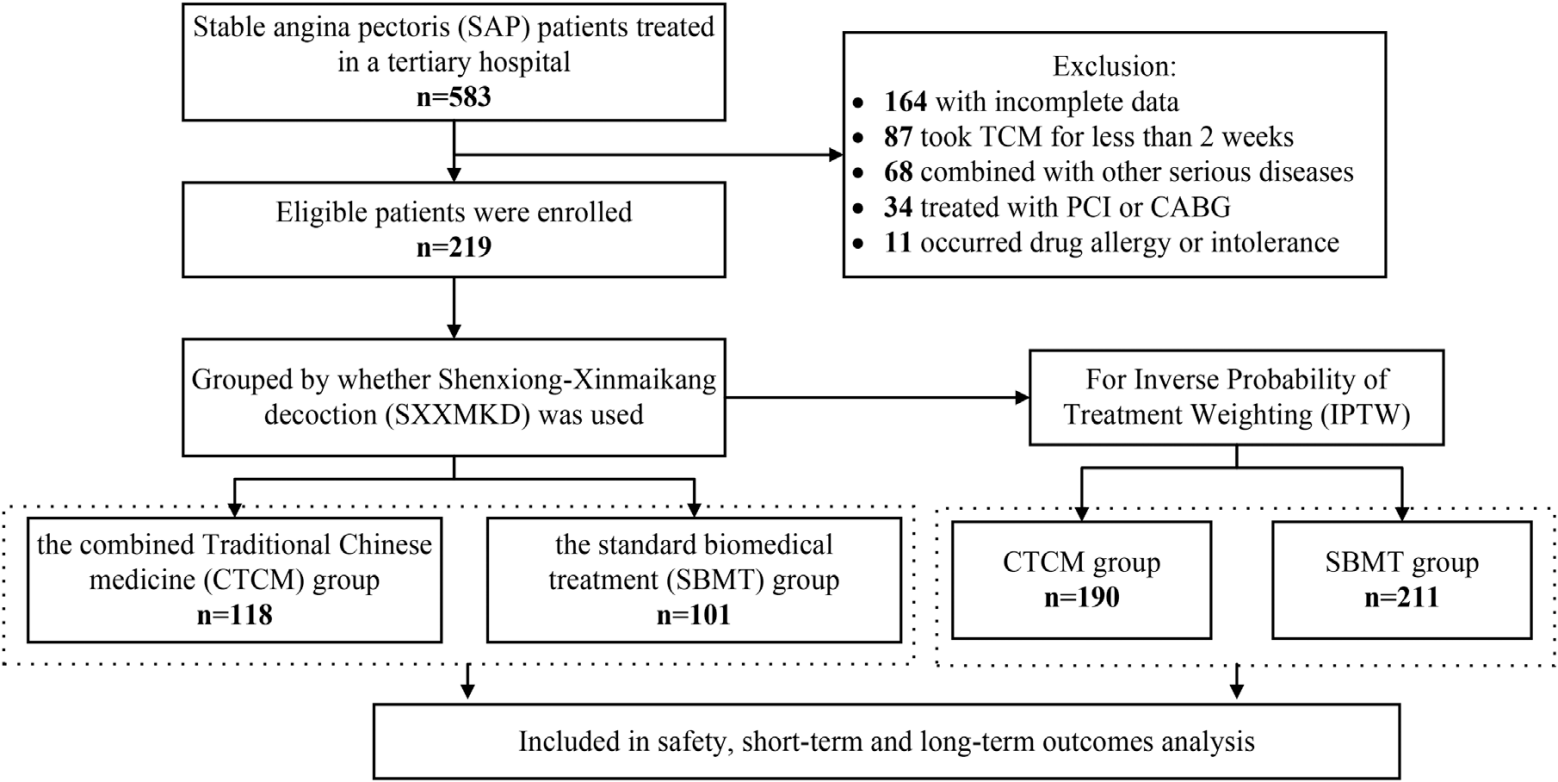
Flowchart of participant selection.

**FIGURE 2:**
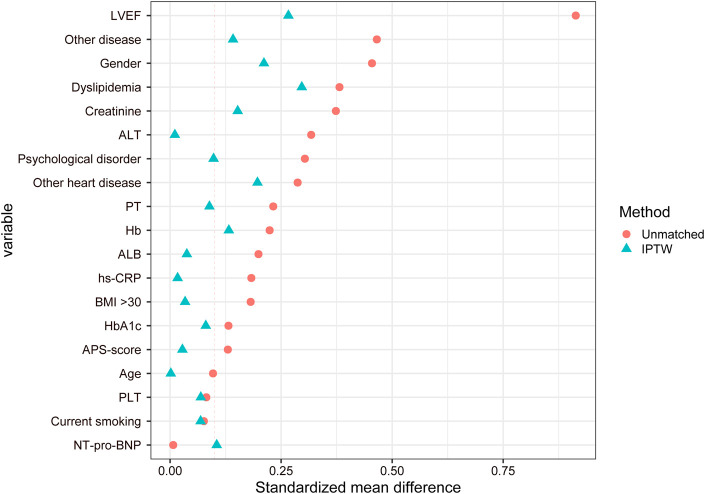
Distribution diagram of the standardized mean difference.

Considering the partial differences in baseline data between the two groups, we utilized the IPTW method for analysis. This approach assigns different weights to each covariate to mitigate potential selection bias in outcomes and other confounding factors in clinical prospective studies, achieving a balanced correction of baseline data. Following the IPTW adjustment, the CTCM group consisted of 190 cases, and the SBMT group comprised 211 cases (Figure 1). The differences in baseline data were reduced to statistically significant differences in gender, dyslipidemia, and LVEF. All other variables showed no statistical significance (P > 0.05), as presented in Table 2. The IPTW significantly balanced the baseline data between the two groups, and the SMD distribution of variables between the groups is illustrated in Figure 2.

**TABLE 2:** Demographic and clinical characteristics of the patients in the two groups before and after IPTW.

Index	Before IPTW				After IPTW			
	CTCM group (*n* = 118)	SBMT group (*n* = 101)	*P*	SMD	CTCM group (*n* = 190)	SBMT group (*n* = 211)	*P*	SMD
Age, years	66.0 (59.8–73.0)	67.0 (59.0–72.0)	0.693	0.097	66.0 (59.0–73.0)	69.0 (60.0–72.0)	0.348	0.002
Gender, Male (n, %)	56 (47.5)	70 (69.3)	0.001	0.455	100 (52.6)	133 (63.0)	0.043	0.211
Comorbid Other Cardiac Diseases^a^	118 (100)	97 (96.0)	0.044	0.287	190 (100)	207 (98.1)	0.125	0.197
Dyslipidemia	110 (93.2)	101 (100)	0.008	0.381	182 (95.8)	211 (100)	0.002	0.297
Psychological Disorders^b^	29 (24.6)	13 (12.9)	0.028	0.303	40 (21.1)	36 (17.1)	0.311	0.098
Smoking History	21 (17.8)	21 (20.8)	0.575	0.076	35 (18.4)	34 (16.1)	0.597	0.069
BMI >30 kg/m^2^ (n, %)	30 (25.4)	34 (33.7)	0.181	0.181	56 (29.5)	59 (28.0)	0.742	0.034
Other Diseases^c^	102 (86.4)	68 (67.3)	0.001	0.466	157 (82.6)	163 (77.3)	0.213	0.142
LVEF (%)			<0.001	0.913			0.011	0.266
<55	4 (3.4)	37 (36.6)			19 (10.0)	41 (19.3)		
≥55	114 (96.6)	64 (63.4)			171 (90.0)	170 (80.7)		
HbA1c (%)			0.334	0.131			0.413	0.080
<6	52 (44.1)	38 (37.6)			77 (40.5)	77 (36.5)		
≥6	66 (55.9)	63 (62.4)			113 (59.5)	134 (63.5)		
NT-pro-BNP (fmol/mL)			0.959	0.007			0.307	0.105
<125	67 (56.8)	57 (56.4)			110 (57.9)	133 (63.0)		
≥125	51 (43.2)	44 (43.6)			80 (42.1)	78 (37.0)		
hs-CRP (mg/L)			0.183	0.183			0.873	0.017
<3	102 (86.4)	93 (92.1)			169 (88.9)	189 (89.6)		
≥3	16 (13.6)	8 (7.9)			21 (11.1)	22 (10.4)		
ALT (IU/L)			0.019	0.318			1.000	0.011
<33	105 (89.0)	78 (77.2)			161 (84.7)	178 (84.4)		
≥33	13 (11.0)	23 (22.8)			29 (15.3)	33 (15.6)		
ALB (g/L)			0.146	0.199			0.798	0.038
<35	28 (23.7)	16 (15.8)			37 (19.5)	38 (18.0)		
≥35	90 (76.3)	85 (84.2)			153 (80.5)	173 (82.0)		
Hb (g/L)			0.102	0.224			0.199	0.132
<120	32 (27.1)	18 (17.8)			41 (21.6)	34 (16.1)		
≥120	86 (72.9)	83 (82.2)			149 (78.4)	177 (83.9)		
PLT (×10^9^/L)			0.549	0.082			0.648	0.069
<125	16 (13.6)	11 (10.9)			25 (13.2)	24 (11.4)		
≥125	102 (86.4)	90 (89.1)			165 (86.8)	187 (88.6)		
PT (s)			0.088	0.232			0.391	0.089
<12.1	82 (69.5)	59 (58.4)			134 (70.5)	140 (66.4)		
≥12.1	36 (30.5)	42 (41.6)			56 (29.5)	71 (33.6)		
Creatinine, Cr (μmol/L)			0.007	0.373			0.134	0.152
<84	52 (44.1)	63 (62.4)			86 (45.3)	112 (53.1)		
≥84	66 (55.9)	38 (37.6)			104 (54.7)	99 (46.9)		
APS-score	7.0 (6.0–11.0)	7.0 (5.0–9.0)	0.491	0.130	7.0 (6.0–9.0)	7.0 (6.0–9.0)	0.369	0.028

^a^ Comorbid Other Cardiac Diseases: This category includes conditions that are associated with hypertension or heart failure, or those who have previously undergone percutaneous coronary intervention (PCI), coronary artery bypass grafting (CAGB), or have a history of using cardiovascular medications.

^b^ Psychological Disorders: This encompasses conditions such as anxiety and depression.

^c^ Other Diseases: Refers to conditions outside the scope of cardiovascular disease, which may include one or more of the following: Chronic Obstructive Pulmonary Disease (COPD), liver disease, chronic renal failure, and diabetes. Variables are presented as mean ± standard deviation (SD), median (Q25, Q75), or number of occurrences (n %).

SD, standard deviation; IPTW, inverse probability of treatment weighting; CTCM, combined traditional Chinese medicine; SBMT, standard biomedical treatment group; SMD, standardized mean difference; BMI, body mass index; LVEF, left ventricular ejection fraction, HbA1c, hemoglobin A1c; NT-pro-BNP, N-terminal–pro-brain natriuretic peptide; hs-CRP, high sensitivity C-reactive protein; ALT, alanine aminotransferase; ALB, albumin; Hb, hemoglobin; PLT, platelet; PT, prothrombin time; APS-score, angina pectoris symptom scoring.

### 3.2 Primary outcome

Patients in both groups showed improvements in angina pectoris symptom score (APS score), lipid indices, CK and hs-CRP after receiving the treatment (Table 3). The intergroup analysis demonstrated that the APS-score for the CTCM group was significantly lower than that of the SBMT group after treatment [5.0 (0–6.0) vs. 5.0 (4.0–7.0), P < 0.001] (Table 4). The findings suggest that SXXMKD is anticipated to decrease the frequency, duration, and intensity of nitroglycerin use in SAP, as well as to reduce the required dosage, thereby offering substantial benefits over the use of standard biomedical treatment alone. Furthermore, the overall effective rate was higher in the CTCM group, at 52.1% (99/190), compared to the SBMT group, at 46.0% (97/211). These findings suggest that integrating TCM with standard biomedical treatment offers distinct benefits in enhancing therapeutic outcomes for angina pectoris.

**TABLE 3:** Comparison of two groups before and after treatment in IPTW cohort.

Group	APS-score	EF (%)	TG (mmol/L)	TC (mmol/L)	LDL-C (mmol/L)	HDL-C (mmol/L)	NT-pro-BNP (fmol/mL)	CK (IU/L)	hs-CRP (mg/L)	cTnI (ng/mL)
*SBMT group*										
Before	7.0 (6.0–9.0)	60.0 (58.0–62.0)	1.40 (1.06–2.16)	4.18 (3.53–4.56)	2.49 (1.56–2.88)	1.10 (0.92–1.38)	100.0 (43.0–257.1)	105.1 (75.1–133.0)	0.85 (0.84–0.90)	0.01 (0.01–0.03)
After	5.0 (4.0–7.0)	61.0 (57.1–62.0)	1.27 (1.02–1.59)	3.66 (3.11–4.33)	2.14 (1.82–2.50)	1.10 (0.99–1.39)	100.0 (50.0–108.0)	81.2 (64.9–115.0)	0.85 (0.55–1.04)	0.01 (0.01–0.03)
*P*	**<0.001**	0.133	**<0.001**	**0.004**	0.912	0.096	**<0.001**	**<0.001**	**0.016**	**0.008**
*CTCM group*										
Before	7.0 (6.0–9.0)	62.0 (60.0–62.0)	1.36 (0.94–2.12)	3.87 (3.55–4.77)	2.08 (1.76–3.00)	1.11 (0.89–1.48)	100.0 (54.0–246.0)	96.5 (73.4–129.6)	0.84 (0.75–1.58)	0.01 (0.01-0.01)
After	5.0 (0–6.0)	62.0 (60.0–63.0)	1.22 (0.98–1.66)	3.69 (3.02–4.50)	1.83 (1.47–2.10)	1.14 (0.91–1.54)	62.8 (50.0–235.5)	71.1 (49.0–100.0)	0.64 (0.57–0.72)	0.01 (0.01–0.02)
*P*	**<0.001**	0.542	**0.041**	**<0.001**	**<0.001**	**<0.001**	0.211	**<0.001**	**<0.001**	0.969

Variables are expressed as mean ± SD, median (Q25, Q75), or n (%). Bold values indicate a statistically significant difference (P < 0.05).

SD: standard deviation; CTCM, combined traditional Chinese medicine; SBMT, standard biomedical treatment; IPTW, inverse probability of treatment weighting; APS-score, angina pectoris symptom scoring; TG, triglyceride; TC, total cholesterol; LDL-C, low-density lipoprotein cholesterol; HDL-C, high-density lipoprotein cholesterol; CK, creatine kinase; cTnI, cardiac troponin I.

**TABLE 4:** Comparison of effectiveness between the two groups in IPTW cohort.

Index	CTCM group (n=190)	SBMT group (n=211)	*P*
Electrocardiogram change			**0.024**
Significantly Effective / Effective	156 (82.1)	153 (72.5)	
Ineffective/Worsened	34 (17.9)	58 (27.5)	
Lipid-lowering efficacy			0.447
Significantly Effective / Effective	136 (71.6)	143 (67.8)	
Ineffectives	54 (28.4)	68 (32.2)	
LVEF (%)	62.0 (60.0-63.0)	61.0 (57.1-62.0)	**0.031**
TG (mmol/L)	1.22 (0.98-1.66)	1.27 (1.02-1.59)	0.413
TC (mmol/L)	3.69 (3.02-4.50)	3.66 (3.11-4.33)	0.543
LDL-C (mmol/L)	1.83 (1.47-2.10)	2.14 (1.82-2.50)	**<0.001**
HDL-C (mmol/L)	1.14 (0.91-1.54)	1.10 (0.99-1.39)	0.332
NT-pro-BNP (fmol/ml)	62.8 (50.0-235.5)	100.0 (50.0-108.0)	0.664
CK (IU/L)	71.1 (49.0-100.0)	81.2 (64.9-115.0)	**<0.001**
hs-CRP (mg/L)	0.64 (0.57-0.72)	0.85 (0.55-1.04)	**0.002**
cTnI (ng/ml)	0.01 (0.01-0.02)	0.01 (0.01-0.03)	**<0.001** ^**a**^
APS-score	5.0 (0-6.0)	5.0 (4.0-7.0)	**<0.001**
Efficacy evaluation of angina pectoris			0.231
Significantly Effective / Effective	99 (52.1)	97 (46.0)	
Ineffective / Worsened	91 (47.9)	114 (54.0)	
Follow-up (months)	20 (15-32)	24.7 (14-34)	0.632
Cardiovascular events (n, %)	62 (32.6)	94 (44.8)	**0.014**
Angina pectoris	33 (17.4)	53 (25.2)	-
Myocardial infarction	28 (14.7)	37 (17.6)	-
Revascularization	1 (0.5)	4 (1.9)	-
All-cause death	0 (0)	0 (0)	-

Variables are expressed as mean ± SD, median (Q25, Q75), or n (%).

SD: standard deviation; CTCM, combined traditional Chinese medicine; SBMT, standard biomedical treatment; IPTW, inverse probability of treatment weighting; LVEF, left ventricular ejection fraction; TG, triglyceride; TC, total cholesterol; LDL-C, low-density lipoprotein cholesterol; HDL-C, high-density lipoprotein cholesterol; CK, creatine kinase; APS-score, angina pectoris symptom scoring; cTnI, cardiac troponin I.

^a^ The mean ± SD, of cTnI for CTCM, group and SBMT, group were 0.01 ± 0.01, 0.03 ± 0.03 ng/mL, respectively.

### 3.3 Secondary outcomes

#### 3.3.1 Electrocardiogram efficacy evaluation

Following the administration of medication, the CTCM group exhibited a significantly higher overall effective rate of 82.1% (156/190) compared to the SBMT group, which had a rate of 72.5% (153/211) (P < 0.05). This statistical difference suggests that integrating Chinese medicine with standard biomedical treatments offers a distinct advantage in enhancing electrocardiogram efficacy compared to standard biomedical treatment alone (Table 4).

#### 3.3.2 Lipid evaluation

Intra-group analysis revealed that low-density lipoprotein (LDL-C) levels were lower and high-density lipoprotein cholesterol (HDL-C) levels were higher in the CTCM group (P < 0.001), but neither changed significantly in the SBMT group (Table 3).

The inter-group comparison after treatment revealed that the LDL-C levels in the CTCM group were significantly reduced compared to the SBMT group [1.83 (1.47–2.10) vs. 2.14 (1.82–2.50) mmol/L, P < 0.001]. No significant differences were observed in the levels of triglycerides (TG), total cholesterol (TC), HDL-C, and the lipid-lowering efficacy rate between the two groups (P > 0.05) (Table 4). The results indicated that the SXXMKD-based treatment was markedly more effective than standard biomedical treatment in reducing blood lipids, particularly in lowering LDL-C levels.

#### 3.3.3 Other indicators

**FIGURE 3:**
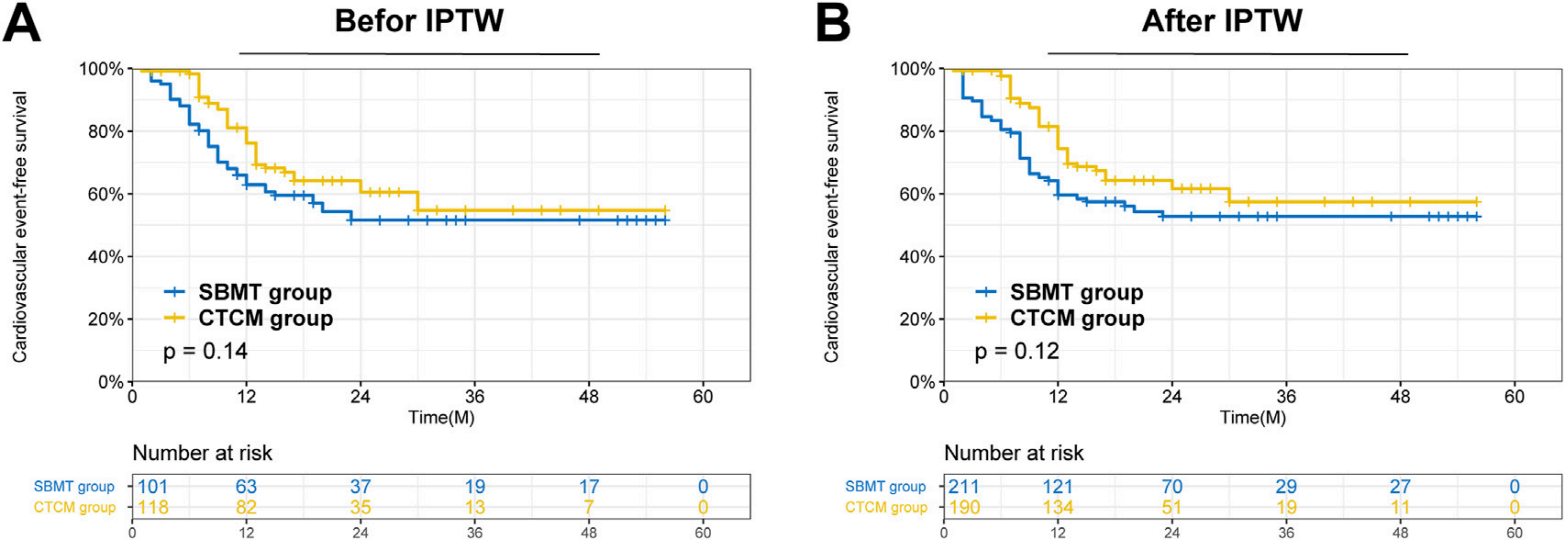
Survival analysis of patients after receiving treatment in the CTCM group versus the SBMT group in the two cohorts. **(A)** Comparison of Cardiovascular events -free survival between the two groups in the entire cohort (*P* = 0.14); **(B)** Comparison of Cardiovascular events -free survival between the two groups in the IPTW cohort (*P* = 0.12).

The CTCM group showed lower CK, hs-CRP, and cTnI levels, and higher LVEF after treatment (all P < 0.05), suggesting that SXXMKD may possess additional anti-inflammatory and cardioprotective effects (Table 4). Long-term results showed that the incidence of cardiovascular events during follow-up was significantly lower in the CTCM group than in the SBMT group (32.6% vs. 44.8%, P = 0.014). There were no deaths, and comparable cardiovascular event-free survival rates were observed in both groups. (Figure 3).

### 3.4 Adverse events

Of the 583 patients initially screened, 11 (1.9%) were excluded due to treatment-related adverse events, with 6 from the combination therapy group and 5 from the standard biomedical treatment group. These events included drug allergies or intolerances, specifically 4 cases of rash and 7 of gastrointestinal symptoms (e.g., nausea, diarrhea). All were managed promptly according to standard medical protocols, including timely adjustment or discontinuation of the medication. No drug-related adverse events were observed in either group (Table 5). After treatment, there was no significant difference between the two groups in terms of ALT, ALB, Hb, PLT and PT (P > 0.05). However, creatinine levels were higher in the SBMT group than in the CTCM group [82.0 (69.9–97.0) vs. 92.0 (64.7–106.0) μmol/L, P = 0.029], suggesting that the use of standard biomedical treatment alone may increase the risk of renal failure.

**TABLE 5:** Safety of SXXMKD in patients with SAP in IPTW cohort.

Index	CTCM group (*n* = 190)	SBMT group (*n* = 211)	*P*
ALT (IU/L)	16.0 (12.0–26.0)	18.0 (14.0–23.0)	0.076
ALB (g/L)	40.5 (37.8–43.4)	40.3 (38.1–42.0)	0.125
Hb (g/L)	131.0 (122.0–145.0)	135.0 (121.1–140.4)	0.476
PLT (×10^9^/L)	228.0 (201.0–276.0)	252.0 (202.0–277.7)	0.142
PT (s)	11.4 (11.0–12.6)	11.5 (11.2–12.0)	0.470
Creatinine (μmol/L)	82.0 (69.9–97.0)	92.0 (64.7–106.0)	**0.029**
Drug-related adverse reactions	0 (0)	0 (0)	-

Variables are expressed as mean ± SD, median (Q25, Q75), or n (%). Bold values indicate a statistically significant difference (*P* < 0.05).

SD: standard deviation; CTCM, combined traditional Chinese medicine; SBMT, standard biomedical treatment; IPTW, inverse probability of treatment weighting; ALT, alanine aminotransferase; ALB, albumin; Hb, hemoglobin; PLT, platelet; PT, prothrombin time.

### 3.5 Sensitivity analysis and subgroup analysis

The results from the entire (unweighted) cohort were included as part of the sensitivity analyses. Additionally, the effectiveness and safety outcomes of the CTCM and SBMT groups were compared across subgroups that were stratified by gender (male or female), the presence of dyslipidemia, and LVEF status (abnormal or normal). Subgroup analysis for patients without dyslipidemia was not performed, as all such cases (n = 8) belonged to the CTCM group. Detailed results are provided in the Supplementary Material (Supplementary Tables S5–S12; Supplementary Figure S3).

## 4 Discussion

This real-world observational study demonstrated that the addition of SXXMKD to standard therapy for stable angina pectoris (SAP) was associated with significant benefits across multiple clinical domains. After adjusting for baseline differences with IPTW, the SXXMKD-based integrative regimen not only improved angina symptoms and electrocardiogram readings but also enhanced cardiac function, modulated lipid profiles, and reduced systemic inflammation. These multifaceted findings suggest that SXXMKD provides a comprehensive therapeutic effect, which can be understood by examining the synergistic actions of its constituent herbs.

A central finding of our study was the significant improvement in symptomatic and functional outcomes. The CTCM group reported a higher overall effective rate on the angina pectoris score (52.1% vs. 46.0%) and showed greater improvements in LVEF, indicating enhanced cardiac contractility and performance. This aligns with the foundational principles of the SXXMKD formula, which is designed based on the TCM theory of “invigorating Qi and activating blood circulation.” This traditional concept is substantiated by modern pharmacology. For instance, key herbs such as Ge Gen and Huang Qi are known to improve myocardial oxygen metabolism, exhibit potent antioxidant activity, and protect cardiomyocytes from oxidative stress, providing a strong mechanistic basis for the functional improvements we observed (Guo et al., 2019; Hao and Xiao, 2020).

Furthermore, our study provides clinical evidence for the anti-inflammatory and anti-atherosclerotic potential of SXXMKD. Patients in the CTCM group exhibited significantly lower levels of LDL-C and the key inflammatory marker hs-CRP. This is crucial, as the oxidation of LDL-C and subsequent uptake by macrophages to form foam cells is a critical initiating event in atherosclerosis (Persson et al., 2008; Zhang et al., 2019). The observed benefits are likely attributable to multiple components. Dan Shen can inhibit acetylated LDL uptake, while the total saponins from San Qi reduce atherosclerotic lesion severity (Li et al., 2022b; Cheng, 2006). Moreover, recent research has highlighted the role of the NLRP3 inflammasome in cardiovascular inflammation. Puerarin, a major component of Ge Gen, has been shown to block this pathway, offering a direct molecular explanation for the reduction in inflammatory markers, such as hs-CRP, seen in our cohort (Zhang et al., 2021).

The analysis of long-term outcomes revealed a nuanced but clinically meaningful picture. While there was no statistically significant difference in overall cardiovascular event-free survival, the incidence of total cardiovascular events was numerically lower in the CTCM group (32.6% vs. 44.8%). More strikingly, we identified a significant early clustering of events in the SBMT group, with 88.3% of their events occurring within the first 12 months, compared to 69.4% in the CTCM group (P = 0.042). This suggests that SXXMKD may confer an early protective effect, delaying the onset of cardiovascular events, even if the cumulative long-term survival curves eventually converge. Regarding safety, the SXXMKD regimen was well-tolerated, with no drug-related adverse events reported. Interestingly, and contrary to concerns that polypharmacy might increase renal burden, our study revealed a potentially nephroprotective effect. Post-treatment serum creatinine levels were significantly lower in the CTCM group. This novel finding may be linked to components like Dan Shen and San Qi, which have been reported to improve renal microcirculation and reduce glomerulosclerosis. This underscores the potential renal safety and even benefit of this integrative therapy when applied appropriately.

Our findings contribute to the growing body of high-quality evidence supporting integrative TCM therapies for cardiovascular diseases. In terms of symptomatic relief, our results, which show improved angina scores and ECG readings, are consistent with a meta-analysis on Danhong injection. That meta-analysis similarly concluded that adjunctive TCM therapy significantly alleviates angina frequency and severity in SAP (Wang et al., 2023; Liu et al., 2021). These results suggest that multiple TCM formulations, including SXXMKD, have a similar beneficial effect on the primary symptoms of stable angina. Beyond symptom control, our findings of improved cardiac function and long-term outcomes align with trends observed in landmark clinical trials of other proprietary Chinese medicines. For example, the study showed that Qishen Yiqi Dripping Pills could significantly improve LVEF in patients with chronic heart failure. This provides strong support for the LVEF enhancement observed in our study cohort (Mao et al., 2020). Additionally, while our observational study lacked the power to definitively assess hard endpoints, the observed trend of delayed early cardiovascular events is conceptually supported by the recent CTS-AMI trial. This large-scale randomized controlled trial (RCT) showed that Tongxinluo capsules significantly reduced the risk of long-term major adverse cardiovascular events in patients with a history of myocardial infarction, affirming the potential of TCM to favorably modulate the long-term prognosis of coronary heart disease (Yang et al., 2023). Our study builds upon this existing evidence by providing the first clinical data for the SXXMKD formula and uncovering novel findings, such as its potential nephroprotective effects. This enriches the evidence base for integrative cardiology.

This study has several limitations that must be acknowledged. First, as a single-center observational study with a relatively small sample size (*n* = 219), its external validity may be limited. The study population was exclusively Chinese, and the applicability of these findings to other racial or ethnic groups requires further investigation. Second, despite the use of IPTW to balance covariates, residual confounding factors inherent to observational designs cannot be entirely eliminated. The robustness of our statistical inferences could be affected by any remaining imbalances. Third, the lack of a universally standardized efficacy scoring system for angina pectoris complicates direct comparisons with other studies. Treatment in real-world TCM practice is also highly personalized, which may affect the generalizability of our standardized protocol. Finally, the variable follow-up durations among participants could introduce time-related biases in survival analysis, which necessitates a cautious interpretation of the long-term outcome data, as discussed. Therefore, multi-center, large-scale, randomized controlled trials are warranted to confirm our findings.

## 5 Conclusion

In conclusion, this real-world study provides compelling evidence that SXXMKD, as an adjunct to standard biomedical treatment, is a safe and effective therapy for patients with stable angina. It not only alleviates symptoms and improves cardiac function but also favorably modulates key lipid and inflammatory pathways. Furthermore, it may offer early protection against cardiovascular events and unexpected renal benefits. These findings support the integration of SXXMKD into the clinical management of SAP and provide a strong rationale for future large-scale trials to validate its efficacy.

## Data Availability

The raw data supporting the conclusions of this article will be made available by the authors, without undue reservation.
