# Supplementary material for: Efficacy and safety of Shenxiong-Xinmaikang Decoction in patients with stable angina pectoris: a real-world prospective observational study
Source: Front Pharmacol. 2025 Jul 30;16:1591959. doi: 10.3389/fphar.2025.1591959 (PMC12344418; doi:10.3389/fphar.2025.1591959)
Supplement: Supplementary file 1 [file Supplementaryfile1.docx]

Supplementary Material

# Supplementary Tables

## Supplementary Table 1

**Supplementary Table 1.** Angina pectoris symptom scoring.

| **Symptom Category** | **Description** | **Score** |
| --- | --- | --- |
| Attack Frequency | None | 0 |
|  | Weekly | 2 |
|  | 1-3 times/day; or Angina Grade II | 4 |
|  | >4 times/day; or Angina Grade III | 6 |
| Duration | None | 0 |
|  | ≤5 minutes | 1 |
|  | >5 min and <10 min | 2 |
|  | ≥10 minutes | 3 |
| Severity | None | 0 |
|  | Not severe, no impact on daily life | 1 |
|  | Severe, requires nitroglycerin | 2 |
|  | Severe episodes affecting daily activities (e.g., dressing, defecation) | 3 |
| Nitroglycerin Usage | None | 0 |
|  | 1-4 tablets/week | 1 |
|  | 5-9 tablets/week | 2 |
|  | >10 tablets/week | 3 |

## Supplementary Table 2

**Supplementary Table 2.** Evaluation of curative effect on angina pectoris symptoms.

| Significantly Effective | Symptoms disappear or almost disappear, n ≥ 70% |
| --- | --- |
| Effective | Frequency, severity, and duration of pain attacks are significantly reduced, 30% ≤ n ≤ 70% |
| Ineffective | Symptoms remain the same as before treatment, 0 ≤ n < 30% |
| Worsened | Frequency, severity, and duration of pain attacks are all increased, n < 0 |

n= (Pre-medication score - Post-medication score) / Post-medication score x 100%

Total effective rate= (Number of significantly effective cases + Number of effective cases) / Total number of cases * 100%

## Supplementary Table 3

**Supplementary Table 3.** Efficacy criteria of electrocardiogram.

| Significantly Effective | Electrocardiogram (ECG) returns to "approximately normal" or reaches "normal ECG". |  |
| --- | --- | --- |
| Effective | ST-segment depression improves by more than 0.05mV after treatment but does not reach normal levels. In the main leads, inverted T-wave changes become shallower (by more than 25%), or T-waves change from flat to upright, and there is improvement in atrioventricular or intraventricular conduction block. | |
| Ineffective | ECG remains essentially the same as before treatment. |  |
| Worsened | ST-segment depression is more than 0.05mV lower than before treatment. In the main leads, inverted T-waves deepen (by more than 25%) or upright T-waves become flat, flat T-waves become inverted, and there is the appearance of ectopic rhythms, atrioventricular conduction block, or intraventricular conduction block. |  |

## Supplementary Table 4

**Supplementary Table 4.** Efficacy criteria of blood lipid.

| Clinical Control | All laboratory tests return to normal. |
| --- | --- |
| Significantly Effective | Lipid profile meets any of the following criteria: Total Cholesterol (TC) decreases by ≥20%, Triglycerides (TG) decrease by ≥40%, High-Density Lipoprotein-Cholesterol (HDL-C) increases by ≥0.26 mmol/L (10 mg/dl). |
| Effective | Lipid profile meets any of the following criteria: TC decreases by ≥10% but <20%, TG decreases by ≥20% but <40%, HDL-C increases by ≥0.104 mmol/L (4 mg/dl) but <0.26 mmol/L (10 mg/dl). |
| Ineffective | Lipid profile does not meet the above criteria. |

## Supplementary Table 5

Supplementary Table 5. Comparison of effectiveness between the two groups of male patients in the IPTW cohort.

| Index | CTCM group (n=100) | SBMT group (n=133) | *P* value |
| --- | --- | --- | --- |
| **Electrocardiogram change** |  |  | **0.011** |
| Significantly Effective / Effective | 78 (78.0) | 83 (62.4) |  |
| Ineffective / Worsened | 22 (22.0) | 50 (37.6) |  |
| **Lipid-lowering efficacy** |  |  | 0.829 |
| Significantly Effective / Effective | 69 (69.0) | 90 (67.7) |  |
| Ineffective | 31 (31.0) | 43 (32.3) |  |
| LVEF (%) | 61.0 (58.2-63.0) | 60.0 (50.1-63.0) | 0.074 |
| TG (mmol/L) | 1.26 (0.98-1.68) | 1.43 (1.08-1.82) | 0.073 |
| TC (mmol/L) | 3.87 (3.35-4.69) | 3.49 (3.07-3.87) | **<0.001** |
| LDL-C (mmol/L) | 1.84 (1.68-2.10) | 1.90 (1.80-2.41) | **<0.001** |
| HDL-C (mmol/L) | 1.14 (0.93-1.55) | 1.06 (0.90-1.25) | **0.001** |
| NT-pro-BNP (fmol/ml) | 52.0 (48.0-170.0) | 100.0 (53.0-133.0) | **0.002** |
| CK (IU/L) | 87.0 (49.0-159.4) | 79.0 (63.1-126.6) | 0.407 |
| hs-CRP (mg/L) | 0.63 (0.58-0.65) | 0.85 (0.50-1.04) | **0.022** |
| cTnI (ng/ml) | 0.01 (0.01-0.02) | 0.01 (0.01-0.03) | **<0.001** |
| APS-score | 4.0 (0-5.1) | 5.0 (4.0-6.0) | **<0.001** |
| **Efficacy evaluation of angina pectoris** |  |  | **<0.001** |
| Significantly Effective / Effective | 70 (70.0) | 63 (47.4) |  |
| Ineffective / Worsened | 30 (30.0) | 70 (52.6) |  |
| **Cardiovascular events (n, %)** | 30 (30.0) | 63 (47.4) | **0.007** |
| Angina pectoris | 12 (12.0) | 34 (25.6) | - |
| Myocardial infarction | 18 (18.0) | 25 (18.8) | - |
| Revascularization | 0 (0) | 4 (3.0) | - |
| **All-cause death** | - | - | - |

Variables are expressed as mean ± SD, median (Q25, Q75), or n (%). SD: standard deviation; CTCM, combined traditional Chinese medicine; SBMT, standard biomedical treatment; IPTW, inverse probability of treatment weighting; LVEF, left ventricular ejection fraction; TG, triglyceride; TC, total cholesterol; LDL-C, low-density lipoprotein cholesterol; HDL-C, high-density lipoprotein cholesterol; CK, creatine kinase; APS-score, angina pectoris symptom scoring; cTnI, cardiac troponin I.

## Supplementary Table 6

Supplementary Table 6. Comparison of effectiveness between the two groups of female patients in the IPTW cohort.

| Index | CTCM group (n=90) | SBMT group (n=78) | *P* value |
| --- | --- | --- | --- |
| **Electrocardiogram change** |  |  | 0.539 |
| Significantly Effective / Effective | 78 (86.7) | 70 (89.7) |  |
| Ineffective / Worsened | 12 (13.3) | 8 (10.3) |  |
| **Lipid-lowering efficacy** |  |  | 0.353 |
| Significantly Effective / Effective | 67 (74.4) | 53 (67.9) |  |
| Ineffective | 23 (25.6) | 25 (32.1) |  |
| LVEF (%) | 62.0 (60.0-63.0) | 61.0 (60.0-62.0) | 0.280 |
| TG (mmol/L) | 1.11 (0.96-1.50) | 1.05 (0.79-1.44) | 0.141 |
| TC (mmol/L) | 3.56 (3.02-4.37) | 4.33 (3.61-4.36) | **<0.001** |
| LDL-C (mmol/L) | 1.68 (1.45-2.12) | 2.36 (1.87-2.87) | **<0.001** |
| HDL-C (mmol/L) | 1.13 (0.88-1.54) | 1.39 (1.23-1.62) | **0.005** |
| NT-pro-BNP (fmol/ml) | 82.6 (52.0-253.0) | 100.0 (9.0-108.0) | **0.011** |
| CK (IU/L) | 66.3 (44.6-89.7) | 86.3 (64.9-105.1) | **<0.001** |
| hs-CRP (mg/L) | 0.65 (0.55-0.78) | 0.85 (0.64-0.92) | **0.030** |
| cTnI (ng/ml) | 0.01 (0.01-0.02) | 0.01 (0.01-0.04) | 0.473 |
| APS-score | 5.0 (4.0-6.0) | 6.0 (4.0-8.0) | **0.007** |
| **Efficacy evaluation of angina pectoris** |  |  | 0.172 |
| Significantly Effective / Effective | 30 (33.3) | 34 (43.6) |  |
| Ineffective / Worsened | 60 (66.7) | 44 (56.4) |  |
| **Cardiovascular events (n, %)** | 31 (34.4) | 30 (38.5) | 0.589 |
| Angina pectoris | 21 (23.3) | 19 (24.4) | - |
| Myocardial infarction | 9 (10.0) | 11 (14.1) | - |
| Revascularization | 1 (1.1) | 0 (0) | - |
| All-cause death | 0 (0) | 0 (0) | - |

Variables are expressed as mean ± SD, median (Q25, Q75), or n (%). SD: standard deviation; CTCM, combined traditional Chinese medicine; SBMT, standard biomedical treatment; IPTW, inverse probability of treatment weighting; LVEF, left ventricular ejection fraction; TG, triglyceride; TC, total cholesterol; LDL-C, low-density lipoprotein cholesterol; HDL-C, high-density lipoprotein cholesterol; CK, creatine kinase; APS-score, angina pectoris symptom scoring; cTnI, cardiac troponin I.

## Supplementary Table 7

Supplementary Table 7. Comparison of effectiveness between the two groups of patients with dyslipidemia in the IPTW cohort.

| Index | CTCM group (n=182) | SBMT group (n=211) | *P* value |
| --- | --- | --- | --- |
| **Electrocardiogram change** |  |  | **0.040** |
| Significantly Effective / Effective | 148 (81.3) | 153 (72.5) |  |
| Ineffective / Worsened | 34 (18.7) | 58 (27.5) |  |
| **Lipid-lowering efficacy** |  |  | 0.433 |
| Significantly Effective / Effective | 130 (71.4) | 143 (67.8) |  |
| Ineffective | 52 (28.6) | 68 (32.2) |  |
| LVEF (%) | 62.0 (60.0-63.0) | 61.0 (57.1-62.0) | **0.032** |
| TG (mmol/L) | 1.22 (0.98-1.68) | 1.27 (1.02-1.59) | 0.713 |
| TC (mmol/L) | 3.68 (3.02-4.50) | 3.66 (3.11-4.33) | 0.642 |
| LDL-C (mmol/L) | 1.83 (1.46-2.10) | 2.14 (1.82-2.50) | **<0.001** |
| HDL-C (mmol/L) | 1.13 (0.91-1.51) | 1.10 (0.99-1.39) | 0.509 |
| NT-pro-BNP (fmol/ml) | 60.8 (50.0-188.0) | 100.0 (50.0-108.0) | 0.250 |
| CK (IU/L) | 71.1 (49.0-100.0) | 81.2 (64.9-115.0) | **0.001** |
| hs-CRP (mg/L) | 0.64 (0.60-0.74) | 0.85 (0.55-1.04) | **0.005** |
| cTnI (ng/ml) | 0.01 (0.01-0.02) | 0.01 (0.01-0.03) | **<0.001** |
| APS-score | 5.0 (0-6.0) | 5.0 (4.0-7.0) | **<0.001** |
| **Efficacy evaluation of angina pectoris** |  |  | 0.096 |
| Significantly Effective / Effective | 99 (54.4) | 97 (46.0) |  |
| Ineffective / Worsened | 83 (45.6) | 114 (54.0) |  |
| **Cardiovascular events (n, %)** | 60 (33.0) | 94 (44.5) | **0.019** |
| Angina pectoris | 33 (18.1) | 53 (25.1) | - |
| Myocardial infarction | 27 (14.9) | 37 (17.5) | - |
| Revascularization | 0 (0) | 4 (1.9) | - |
| **All-cause death** | 0 (0) | 0 (0) | - |

Variables are expressed as mean ± SD, median (Q25, Q75), or n (%). SD: standard deviation; CTCM, combined traditional Chinese medicine; SBMT, standard biomedical treatment; IPTW, inverse probability of treatment weighting; LVEF, left ventricular ejection fraction; TG, triglyceride; TC, total cholesterol; LDL-C, low-density lipoprotein cholesterol; HDL-C, high-density lipoprotein cholesterol; CK, creatine kinase; APS-score, angina pectoris symptom scoring; cTnI, cardiac troponin I.

## Supplementary Table 8

Supplementary Table 8. Comparison of effectiveness between the two groups of patients with abnormal LVEF in the IPTW cohort.

| Index | CTCM group (n=19) | SBMT group (n=41) | *P* value |
| --- | --- | --- | --- |
| **Electrocardiogram change** |  |  | **0.001** |
| Significantly Effective / Effective | 0 (0) | 16 (39.0) |  |
| Ineffective / Worsened | 19 (100) | 25 (61.0) |  |
| **Lipid-lowering efficacy** |  |  | **0.012** |
| Significantly Effective / Effective | 19 (100) | 29 (70.7) |  |
| Ineffective | 0 (0) | 12 (29.3) |  |
| LVEF (%) | 49.0 (49.0-50.0) | 50.0 (41.7-50.0) | 0.896 |
| TG (mmol/L) | 1.60 (0.83-1.60) | 1.58 (1.40-1.82) | 0.256 |
| TC (mmol/L) | 4.69 (2.82-4.69) | 3.82 (3.17-4.35) | 0.626 |
| LDL-C (mmol/L) | 2.10 (1.49-2.10) | 2.15 (1.80-2.50) | **0.003** |
| HDL-C (mmol/L) | 1.05 (0.81-1.05) | 1.09 (0.97-1.34) | **<0.001** |
| NT-pro-BNP (fmol/ml) | 12.0 (12.0-369.0) | 90.0 (6.3-264.7) | 0.417 |
| CK (IU/L) | 198.7 (56.4-198.7) | 93.2 (63.1-146.0) | 0.652 |
| hs-CRP (mg/L) | 0.60 (0.60-1.59) | 0.99 (0.50-1.13) | 0.134 |
| cTnI (ng/ml) | 0.01 (0.01-0.07) | 0.02 (0.01-0.07) | 0.068 |
| APS-score | 0.0 (0-6.0) | 4.0 (4.0-5.0) | 0.085 |
| **Efficacy evaluation of angina pectoris** |  |  | 0.630 |
| Significantly Effective / Effective | 11 (57.9) | 21 (51.2) |  |
| Ineffective / Worsened | 8 (42.1) | 20 (48.8) |  |
| **Cardiovascular events (n, %)** | 8 (42.1) | 24 (58.5) | 0.235 |
| Angina pectoris | 4 (21.1) | 17 (41.5) | - |
| Myocardial infarction | 4 (21.1) | 7 (17.0) | - |
| Revascularization | 0 (0) | 0 (0) | - |
| **All-cause death** | 0 (0) | 0 (0) | - |

Variables are expressed as mean ± SD, median (Q25, Q75), or n (%). SD: standard deviation; CTCM, combined traditional Chinese medicine; SBMT, standard biomedical treatment; IPTW, inverse probability of treatment weighting; LVEF, left ventricular ejection fraction; TG, triglyceride; TC, total cholesterol; LDL-C, low-density lipoprotein cholesterol; HDL-C, high-density lipoprotein cholesterol; CK, creatine kinase; APS-score, angina pectoris symptom scoring; cTnI, cardiac troponin I.

## Supplementary Table 9

Supplementary Table 9. Comparison of effectiveness between the two groups of patients with normal LVEF in the IPTW cohort.

| Index | CTCM group (n=171) | SBMT group (n=170) | *P* value |
| --- | --- | --- | --- |
| **Electrocardiogram change** |  |  | **0.005** |
| Significantly Effective / Effective | 156 (91.2) | 137 (80.6) |  |
| Ineffective / Worsened | 15 (8.8) | 33 (19.4) |  |
| **Lipid-lowering efficacy** |  |  | 0.700 |
| Significantly Effective / Effective | 118 (69.0) | 114 (67.1) |  |
| Ineffective | 53 (31.0) | 56 (32.9) |  |
| LVEF (%) | 62.0 (60.0-63.0) | 61.5 (60.0-63.0) | **0.223** |
| TG (mmol/L) | 1.22 (0.99-1.76) | 1.25 (0.99-1.76) | 0.867 |
| TC (mmol/L) | 3.69 (3.05-4.50) | 3.66 (3.08-4.33) | 0.443 |
| LDL-C (mmol/L) | 1.80 (1.43-2.12) | 2.04 (1.82-2.50) | **<0.001** |
| HDL-C (mmol/L) | 1.23 (0.92-1.58) | 1.10 (0.99-1.39) | 0.073 |
| NT-pro-BNP (fmol/ml) | 64.3 (50.0-190.5) | 100.0 (53.0-108.0) | 0.391 |
| CK (IU/L) | 71.1 (49.0-95.6) | 79.3 (65.2-111.6) | **<0.001** |
| hs-CRP (mg/L) | 0.64 (0.55-0.71) | 0.85 (0.64-0.92) | **<0.001** |
| cTnI (ng/ml) | 0.01 (0.01-0.02) | 0.01 (0.01-0.02) | **0.001** |
| APS-score | 5.0 (0-6.0) | 6.0 (4.0-7.0) | **<0.001** |
| **Efficacy evaluation of angina pectoris** |  |  | 0.212 |
| Significantly Effective / Effective | 88 (51.5) | 76 (44.7) |  |
| Ineffective / Worsened | 83 (48.5) | 94 (55.3) |  |
| **Cardiovascular events (n, %)** | 54 (31.6) | 70 (41.2) | 0.065 |
| Angina pectoris | 29 (17.0) | 36 (21.2) | - |
| Myocardial infarction | 24 (14.0) | 30 (17.6) | - |
| Revascularization | 1 (0.6) | 4 (2.4) | - |
| **All-cause death** | 0 (0) | 0 (0) | - |

Variables are expressed as mean ± SD, median (Q25, Q75), or n (%). SD: standard deviation; CTCM, combined traditional Chinese medicine; SBMT, standard biomedical treatment; IPTW, inverse probability of treatment weighting; LVEF, left ventricular ejection fraction; TG, triglyceride; TC, total cholesterol; LDL-C, low-density lipoprotein cholesterol; HDL-C, high-density lipoprotein cholesterol; CK, creatine kinase; APS-score, angina pectoris symptom scoring; cTnI, cardiac troponin I.

## Supplementary Table 10

Supplementary Table 10. Safety of SXXMKD in patients with SAP in each subgroup cohort.

| Index | CTCM group | SBMT group | *P* |
| --- | --- | --- | --- |
| *Male patients* | *n=100* | *n=133* |  |
| ALT (IU/L) | 20.0 (14.0-36.0) | 18.0 (13.0-23.4) | **0.034** |
| ALB (g/L) | 40.7 (38.0-43.7) | 40.2 (37.9-42.0) | **0.013** |
| Hb (g/L) | 142.0 (129.0-151.0) | 135.0 (124.0-142.0) | **<0.001** |
| PLT (G/L) | 225.0 (201.0-269.0) | 251.6 (183.0-276.5) | 0.565 |
| PT (s) | 11.3 (11.1-12.0) | 11.4 (11.1-12.0) | 0.178 |
| creatinine (μmol/L) | 87.5 (78.0-100.0) | 100.0 (86.4-115.0) | **<0.001** |
| Drug-related adverse reactions | 0 (0) | 0 (0) | - |
| *Female patients* | *n=90* | *n=78* |  |
| ALT (IU/L) | 14.8 (12.0-17.1) | 18.6 (14.0-23.0) | **<0.001** |
| ALB (g/L) | 40.3 (37.2-43.4) | 40.5 (39.1-42.4) | 0.535 |
| Hb (g/L) | 123.4 (113.0-133.0) | 130.0 (119.0-135.0) | **0.025** |
| PLT (G/L) | 237.0 (190.5-277.4) | 259.0 (225.0-282.0) | 0.108 |
| PT (s) | 11.5 (11.0-12.9) | 11.5 (11.2-12.2) | 0.897 |
| creatinine (μmol/L) | 71.0 (61.2-85.0) | 62.0 (58.0-84.0) | **0.017** |
| Drug-related adverse reactions | 0 (0) | 0 (0) |  |
| *Patients with dyslipidemia* | *n=182* | *n=211* |  |
| ALT (IU/L) | 17.0 (12.0-26.0) | 18.0 (14.0-23.0) | 0.192 |
| ALB (g/L) | 40.5 (37.9-43.4) | 40.3 (38.1-42.0) | 0.108 |
| Hb (g/L) | 133.0 (123.0-145.1) | 135.0 (121.1-140.4) | 0.286 |
| PLT (G/L) | 229.0 (201.0-278.0) | 252.0 (202.0-277.7) | 0.309 |
| PT (s) | 11.4 (11.1-12.3) | 11.5 (11.2-12.0) | 0.475 |
| creatinine (μmol/L) | 82.0 (70.0-97.0) | 92.0 (64.7-106.0) | **0.031** |
| Drug-related adverse reactions | 0 (0) | 0 (0) | - |
| *Patients with abnormal LVEF* | *n=19* | *n=41* |  |
| ALT (IU/L) | 38.0 (12.0-38.0) | 21.0 (14.0-26.6) | 0.255 |
| ALB (g/L) | 43.4 (40.3-43.4) | 39.1 (37.4-40.5) | **0.001** |
| Hb (g/L) | 148.0 (113.0-148.0) | 133.0 (120.3-135.0) | 0.986 |
| PLT (G/L) | 201.0 (129.0-201.0) | 256.0 (200.0-305.0) | **<0.001** |
| PT (s) | 11.1 (11.1-23.2) | 11.5 (11.3-12.3) | 0.957 |
| creatinine (μmol/L) | 102.0 (82.0-102.0) | 104.6 (83.0-126.6) | 0.074 |
| Drug-related adverse reactions | 0 (0) | 0 (0) | - |
| *Patients with normal LVEF* | *n=171* | *n=170* |  |
| ALT (IU/L) | 15.8 (12.0-25.0) | 18.0 (14.0-23.0) | **0.036** |
| ALB (g/L) | 40.5 (37.2-43.6) | 40.5 (38.2-42.0) | 0.586 |
| Hb (g/L) | 148.0 (113.0-148.0) | 133.0 (120.3-135.0) | 0.825 |
| PLT (G/L) | 236.0 (208.0-278.0) | 247.0 (212.0-268.0) | 0.933 |
| PT (s) | 11.4 (10.8-12.3) | 11.4 (11.1-12.0) | 0.583 |
| creatinine (μmol/L) | 82.0 (69.0-92.0) | 89.0 (62.0-101.0) | 0.165 |
| Drug-related adverse reactions | 0 (0) | 0 (0) | - |

Variables are expressed as mean ± SD, median (Q25, Q75), or n (%). SD: standard deviation; CTCM, combined traditional Chinese medicine; SBMT, standard biomedical treatment; IPTW, inverse probability of treatment weighting; ALT, alanine aminotransferase; ALB, albumin; Hb, hemoglobin; PLT, platelet; PT, prothrombin time.

## Supplementary Table 11

Supplementary Table 11. Comparison of effectiveness between the two groups in the entire cohort.

| Index | CTCM group (n=118) | SBMT group (n=101) | *P* value |
| --- | --- | --- | --- |
| **Electrocardiogram change** |  |  | **<0.001** |
| Significantly Effective / Effective | 105 (89.0) | 67 (66.3) |  |
| Ineffective / Worsened | 13 (11.0) | 34 (33.7) |  |
| **Lipid-lowering efficacy** |  |  | 0.377 |
| Significantly Effective / Effective | 80 (67.8) | 74 (73.3) |  |
| Ineffective | 38 (32.2) | 27 (26.7) |  |
| LVEF (%) | 62.0 (60.0-63.0) | 60.0 (50.0-62.0) | **<0.001** |
| TG (mmol/L) | 1.22 (0.98-1.75) | 1.43 (0.97-1.63) | 0.390 |
| TC (mmol/L) | 3.68 (3.02-4.50) | 3.60 (3.08-4.17) | 0.475 |
| LDL-C (mmol/L) | 1.83 (1.47-2.12) | 2.05 (1.75-2.41) | **<0.001** |
| HDL-C (mmol/L) | 1.16 (0.91-1.52) | 1.10 (1.01-1.38) | 0.509 |
| NT-pro-BNP (fmol/ml) | 61.0 (50.0-106.5) | 100.0 (50.0-106.5) | 0.383 |
| CK (IU/L) | 71.1 (49.0-95.5) | 88.3 (66.0-126.6) | **<0.001** |
| hs-CRP (mg/L) | 0.64 (0.55-0.72) | 0.85 (0.50-1.06) | **0.027** |
| cTnI (ng/ml) | 0.01 (0.01-0.02) | 0.02 (0.01-0.06) | **<0.001** |
| APS-score | 5.0 (0-6.0) | 5.0 (4.0-6.0) | **0.026** |
| **Efficacy evaluation of angina pectoris** |  |  | 0.342 |
| Significantly Effective / Effective | 59 (50.0) | 44 (43.6) |  |
| Ineffective / Worsened | 59 (50.0) | 57 (56.4) |  |
| **Cardiovascular events (n, %)** | 40 (33.9) | 46 (45.5) | 0.079 |
| Angina pectoris | 23 (19.5) | 25 (24.8) | - |
| Myocardial infarction | 16 (13.6) | 18 (17.8) | - |
| Revascularization | 1 (0.8) | 3 (2.9) | - |
| **All-cause death** | - | - | - |

Variables are expressed as mean ± SD, median (Q25, Q75), or n (%). SD: standard deviation; CTCM, combined traditional Chinese medicine; SBMT, standard biomedical treatment; LVEF, left ventricular ejection fraction; TG, triglyceride; TC, total cholesterol; LDL-C, low-density lipoprotein cholesterol; HDL-C, high-density lipoprotein cholesterol; CK, creatine kinase; APS-score, angina pectoris symptom scoring; cTnI, cardiac troponin I.

## Supplementary Table 12

Supplementary Table 12. Safety of SXXMKD in patients with SAP in the entire cohort.

| Index | CTCM group (n=118) | SBMT group (n=101) | *P* |
| --- | --- | --- | --- |
| ALT (IU/L) | 15.0 (12.0-22.5) | 19.0 (14.5-25.0) | **0.018** |
| ALB (g/L) | 40.5 (37.6-43.5) | 40.3 (38.3-42.7) | 0.645 |
| Hb (g/L) | 129.5 (122.0-142.0) | 135.0 (121.0-142.0) | 0.595 |
| PLT (G/L) | 226.0 (199.3-270.8) | 248.0 (195.0-286.0) | 0.191 |
| PT (s) | 11.4 (10.8-12.8) | 11.5 (11.2-12.2) | 0.228 |
| creatinine (μmol/L) | 82.5 (69.5-93.8) | 87.2 (67.0-106.5) | 0.116 |
| Drug-related adverse reactions | 0 (0) | 0 (0) | - |

Variables are expressed as mean ± SD, median (Q25, Q75), or n (%). SD: standard deviation; CTCM, combined traditional Chinese medicine; SBMT, standard biomedical treatment; ALT, alanine aminotransferase; ALB, albumin; Hb, hemoglobin; PLT, platelet; PT, prothrombin time.

# Supplementary Figures

## Supplementary Figure 1


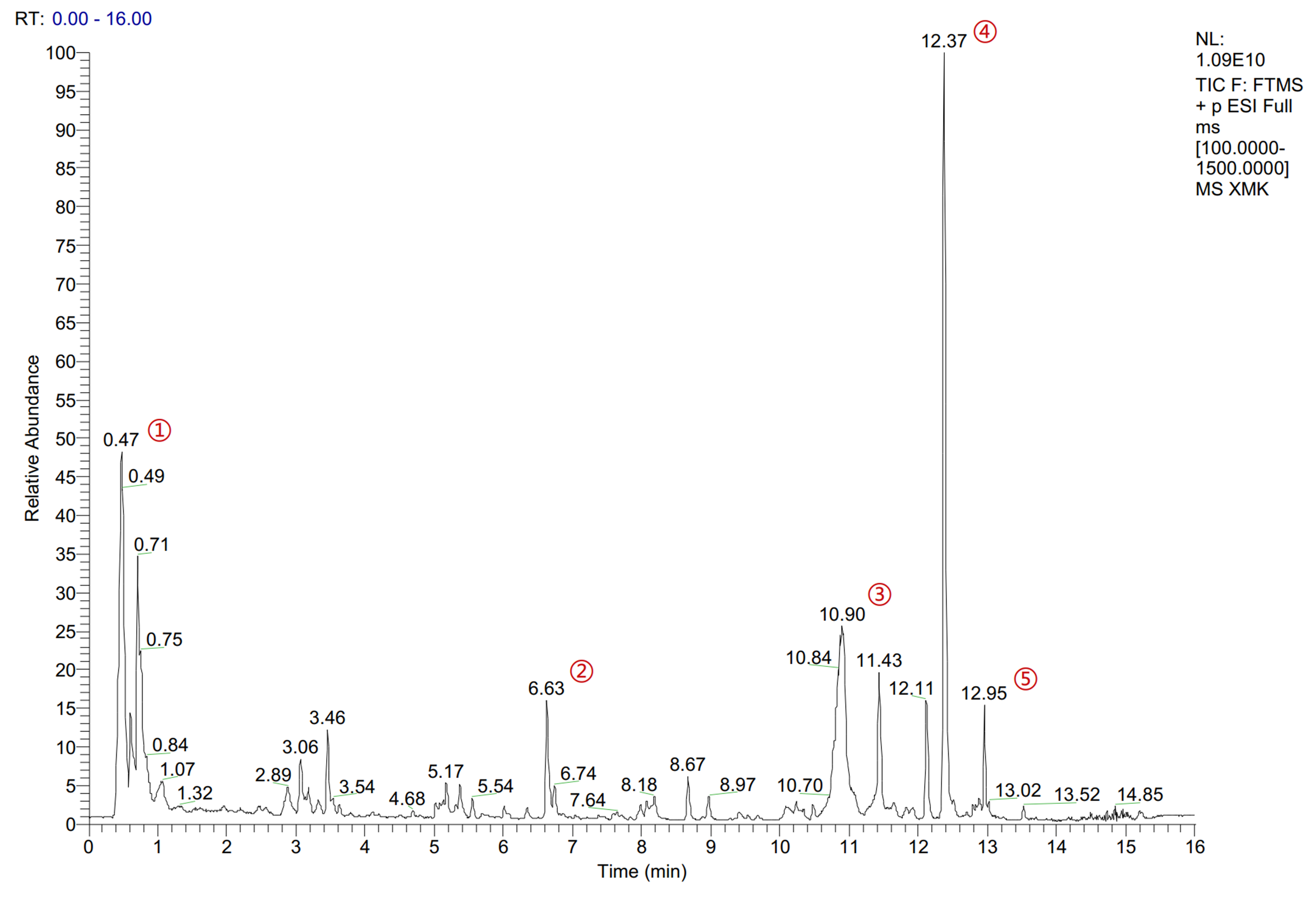


**Supplementary Figure 1.** Mass Spectra of SXXMKD in Positive Ion Mode.

1. RT: 0.47 min - Puerarin, from Ge Gen (*Pueraria lobata (Willd.) Qhwi*), m/z: 417.12, relative abundance: 1.4%.
2. RT: 6.63 min - Salvianolic acid B, from Hong Jing Tian (*Rhodiola rosea L.*), m/z: 719.18, relative abundance: 1.0%.
3. RT: 10.90 min - Ferulic acid, from Chuan Xiong (*Ligusticum chuanxiong Hort.*), m/z: 195.07, relative abundance: 2.4%.
4. RT: 12.37 min - Notoginsenoside R1, from San Qi (*Panax notoginseng (Burkill) F.H.Chen*), m/z: 801.48, relative abundance: 100% (Base Peak).
5. RT: 12.95 min - Calycosin, from Huang Qi (*Astragalus membranaceus (Fisch.) Bunge*), m/z: 285.08, relative abundance: 3.9%.

## Supplementary Figure 2


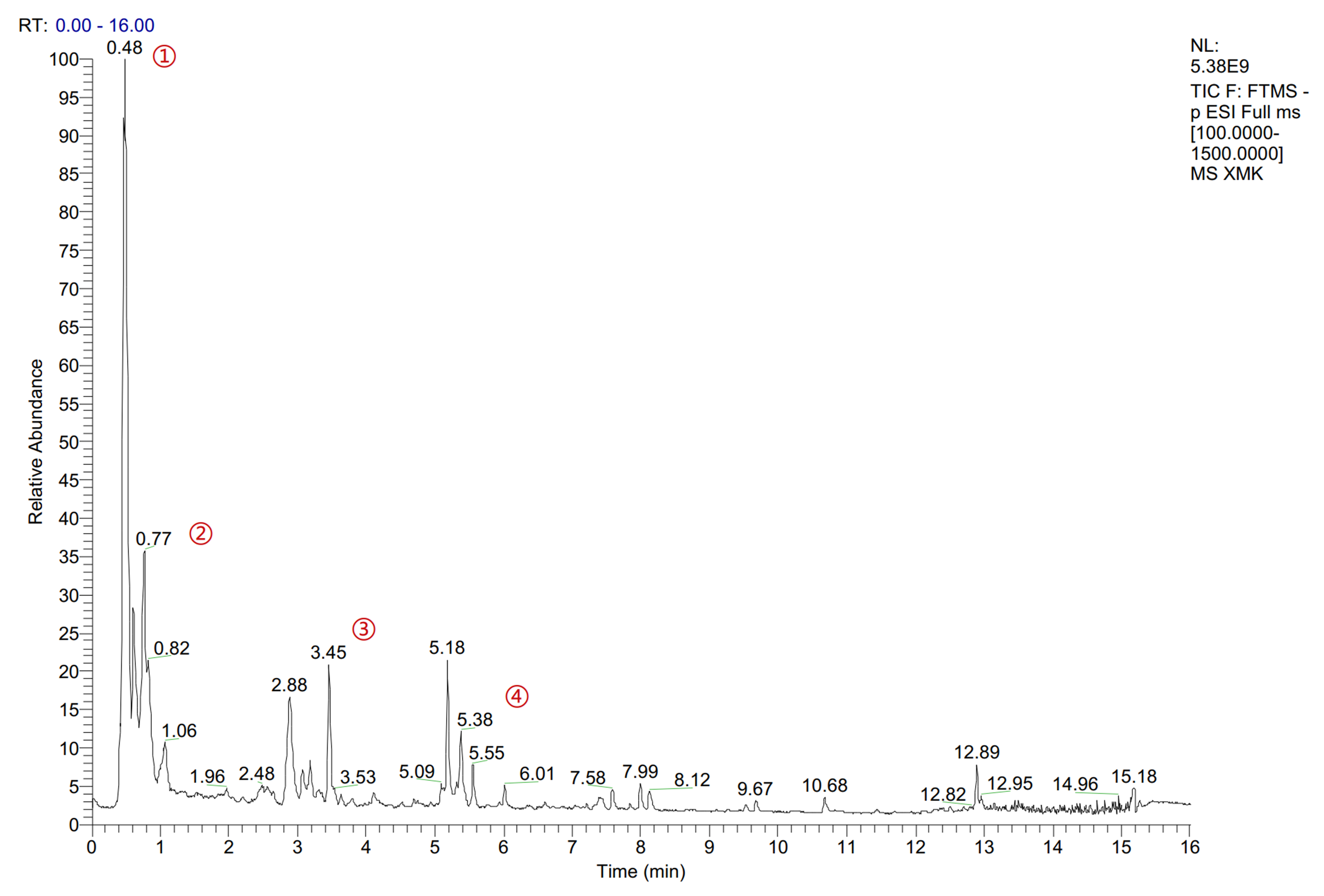


**Supplementary Figure 2**. Mass Spectra of SXXMKD in Negative Ion Mode.

1. RT: 0.48 min - Danshensu, from Dan Shen (*Salvia miltiorrhiza Bunge*), m/z: 197.05, relative abundance: 100% (Base Peak).
2. RT: 0.77 min - Protocatechuic aldehyde, from Dan Shen (*Salvia miltiorrhiza Bunge*), m/z: 137.02, relative abundance: 26.0%.
3. RT: 3.45 min - Salvianolic acid A, from Dan Shen (*Salvia miltiorrhiza Bunge*), m/z: 493.11, relative abundance: 17.4%.
4. RT: 5.38 min - Rosmarinic acid, from Dan Shen (*Salvia miltiorrhiza Bunge*), m/z: 359.08, relative abundance: 19.5%.

## Supplementary Figure 3


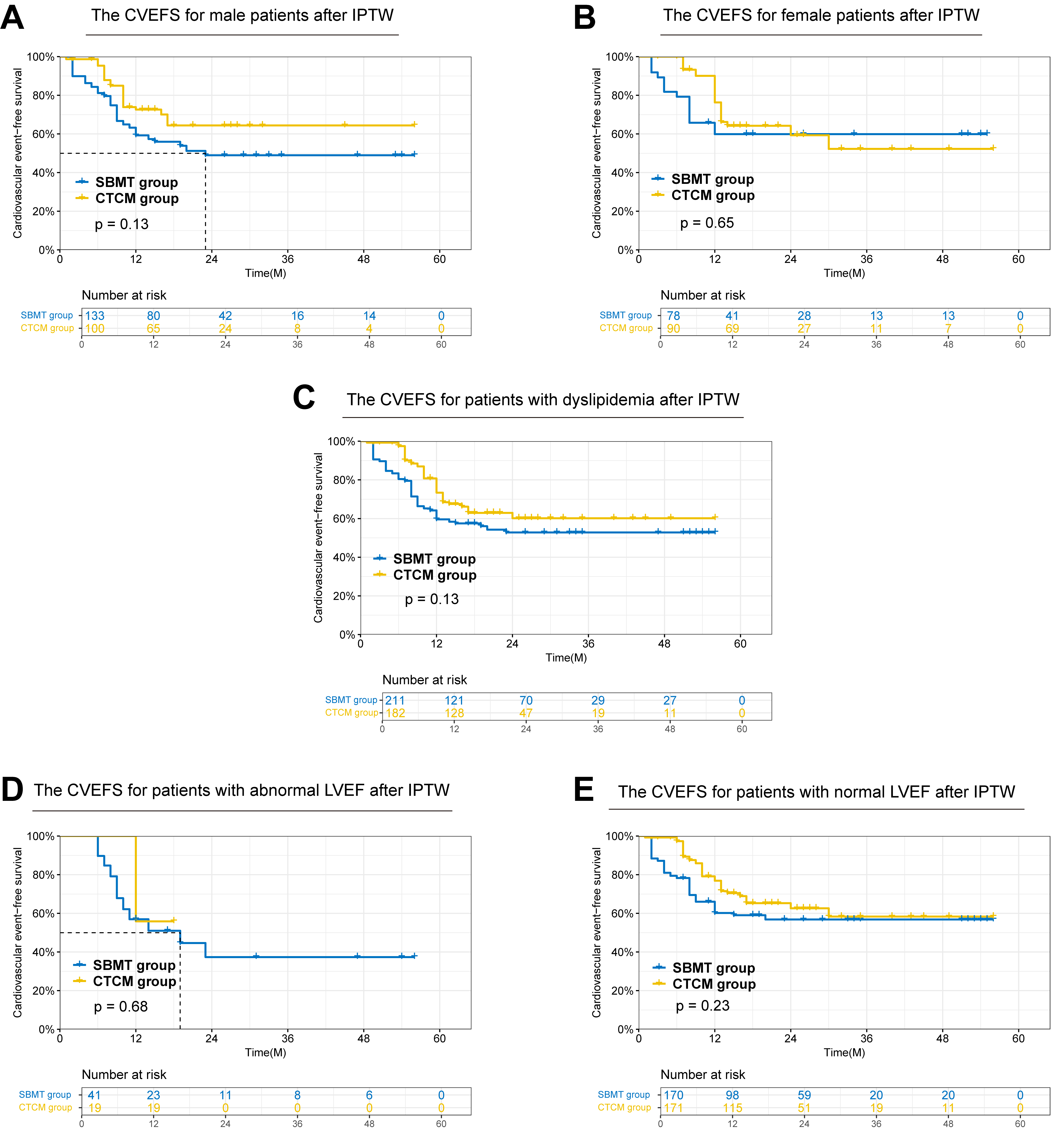


**Supplementary Figure 3.** Subgroup analysis of CVEFS between the CTCM and SBMT groups in patients with stable angina pectoris after treatment. (A, B) CVEFS comparison between groups for male and female patients after IPTW; (C) CVEFS comparison for patients with dyslipidemia after IPTW; (D, E) CVEFS comparison for patients with abnormal and normal LVEF after IPTW. CVEFS, cardiovascular event-free survival; CTCM, combined Traditional Chinese medicine; SBMT, standard biomedical treatment; IPTW, inverse probability of treatment weighting; 3D: three-dimensional; LVEF, left ventricular ejection fraction.

# Supplementary Methods

## Inverse probability of treatment weighting

Propensity score (PS) methods are commonly used to reduce confounding bias in non-randomized studies by estimating the probability that each individual receives a particular treatment. Based on these scores, individuals can be matched (PSM) or weighted (IPTW) to balance baseline characteristics and simulate the effect of randomization, thereby improving group comparability.

In this study, we used the inverse probability of treatment weighting (IPTW) approach. Each patient was assigned a weight based on the inverse of their estimated propensity score: for patients in the CTCM group, the weight was 1/PS; for those in the SBMT group, it was 1/(1–PS). This method adjusts the contribution of each individual in the analysis, helping to reduce selection bias and unbalanced covariates between groups.

## Data collection

The laboratory data were collected by two medical postgraduates (L.C. and P.Y.) who were blinded to the treatment allocation. In cases of data discrepancies, a third investigator (L.Y.Z.) independently verified the records. Electrocardiogram readings were interpreted by two attending cardiologists (M.J.N. and C.Y.Q.), both of whom were also blinded to group assignments. None of these individuals participated in the subsequent statistical analyses.

## SXXMKD Preparation and Individualized Adjustments

The herbal materials for this study’s formula-Huang Qi (15g), Tai Zi Shen (15g), Shan Zha (15g), Chuan Xiong (10g), Ge Gen (15g), Hong Jing Tian (6g), Dan Shen (15g), Mu Xiang (10g) and San Qi (10g)-were purchased from Guangdong Yuanshengtai Pharmaceutical Co., Ltd. (Guangdong, China). The materials were pulverized and passed through a 60-mesh sieve before use. The preparation process of the decoction is completed under the standardized operating procedures of the hospital's traditional Chinese medicine pharmacy. All herbs, excluding Mu Xiang, were soaked in 10 volumes (v/w) of purified water for 30 minutes. The mixture was brought to a boil and then simmered at (95±2)°C for 20 minutes. Subsequently, Mu Xiang (10g) was added for the final 10 minutes of decoction (late addition method). The dregs were decocted again with 8 volumes of water for 20 minutes. The filtrates from both decoctions were combined and concentrated under reduced pressure at 40°C to a final volume of 400 mL. The final product was packaged into 200 mL bags for patients to take orally twice daily. The preparation and use of this formula comply with the Drug Administration Law of the People’s Republic of China and the regulatory standards for in-hospital preparations.

Personalized modifications were made to address the patient's coexisting symptoms without altering the core SXXMKD formula. The four main adjustments were:

1. Qi and Yin vs. Qi and Blood Deficiency: Codonopsis pilosula was substituted for Tai Zi Shen (same dose) when stronger Qi tonification was needed.
2. Gastrointestinal discomfort: Pericarpium Citri Reticulatae or Dioscorea opposita (both 10 g) were added.
3. Low back or joint pain: Eucommia ulmoides (10 g) or Achyranthes bidentata (5 g) was added.
4. Poor sleep: Semen Ziziphi Spinosae (10 g) or Fructus mume (10 g) was added.

In our study, the SXXMKD used was prepared strictly in accordance with the Pharmacopoeia of the people's Republic of China (2020 Edition), and all raw herbal materials were sourced from certified suppliers and processed under quality-controlled conditions [Guangdong Yuanshengtai Pharmaceutical Co., Ltd. (Guangdong, China)].

According to the Drug Administration Law of the People’s Republic of China and the Regulations for the Administration of In-hospital Preparations, SXXMKD has been formally approved as an in-hospital preparation by the Guangdong Provincial Drug Administration. Its composition, processing, quality control, and safety evaluation were carried out under Good Clinical Practice (GCP) and national regulatory guidelines, ensuring its legal status and preclinical safety prior to clinical use.

## A Priori Safety Assessment of the SXXMKD Formula

The safety profile of the SXXMKD formula was established prior to the initiation of this clinical study based on a multi-faceted evaluation, encompassing historical evidence, literature review, Traditional Chinese Medicine (TCM) theory, and formal regulatory approval.

### Foundation in pharmacopoeia and established clinical use

All nine herbs in the SXXMKD formula are officially listed in the *Pharmacopoeia of the People's Republic of China* (2020 Edition). Each herb possesses a long history of safe clinical application for treating cardiovascular and related conditions, providing a strong foundation for its general safety.

### Literature-based safety profile of key herbs

Some literatures have confirmed the safety of the principal herbs. For instance, Dan Shen and San Qi, the "monarch" drugs in this formula, are widely documented for their cardiovascular benefits.^[1]^ Their primary safety consideration relates to potential anticoagulant effects, which was established as a key monitoring parameter in our clinical protocol.^[2,3]^ Other herbs, such as Huang Qi and Ge Gen, are well-tolerated and classified as food-homologous materials in China, further supporting their safety.^[4]^ No significant toxicity has been reported for the prescribed dosages of the constituent herbs.

Furthermore, the rationale for the SXXMKD formulation is strongly supported by the precedent of well-established herbal combinations used in commercially successful and regulatorily approved medicines for cardiovascular disease in China. The core combination of Dan Shen, San Qi, and Ge Gen forms the therapeutic backbone of our SXXMKD formula. This same evidence-based trio is central to widely used marketed drugs such as Xinkeshu Capsule (which also includes some of our formula's components like Crataegus pinnatifida and Aucklandia lappa) and Xinmaikang Capsule.^[5-8]^ Our SXXMKD builds upon this established foundation by incorporating additional herbs, such as the Qi-tonifying Astragalus membranaceus and Pseudostellaria heterophylla, based on TCM principles to provide a more holistic and balanced therapeutic effect. Concurrently, SXXMKD was intentionally designed as a purely plant-based formula, omitting potent animal-derived constituents like Moschus (Musk) and Calculus Bovis (Cattle Bezoar) found in Xinmaikang Capsule. This strategic modification not only avoids the use of endangered species but also potentially offers a broader safety margin. This approach of rationally modifying a classic, approved combination further substantiates the a priori safety assessment of our formula.

### Prospective Pilot Clinical Safety Study

To supplement literature-based evidence with direct data on the complete formula, a prospective, open-label, single-arm pilot study was conducted in Jan. 2019 under the supervision of the Ethics Committee of Zhujiang Hospital, Southern Medical University, prior to the formula's registration as an in-hospital preparation. The study enrolled ten participants (five patients with stable coronary heart disease and five healthy volunteers), who were administered the SXXMKD formula for two weeks (No. 2018-KY-012-01). Safety was rigorously assessed through the active monitoring of adverse events and the evaluation of hepatic and renal function at baseline, one week after, and one month after the intervention. The results demonstrated that the SXXMKD formula was well-tolerated, with no significant adverse events or clinically relevant changes in liver or kidney function markers observed. Although this pilot study was not published, as its primary purpose was to provide pivotal safety data for the regulatory application, its findings provided critical, prospective human safety evidence.

### Rationale for the herbal combination based on TCM theory

The formulation of SXXMKD is not a random collection of herbs but adheres strictly to the "Jun-Chen-Zuo-Shi" (Monarch, Minister, Assistant, and Messenger) principles of TCM. This structured combination is designed to enhance therapeutic efficacy while mitigating potential side effects through synergistic and antagonistic interactions. This theoretical framework, refined over centuries, provides a rational basis for minimizing adverse interactions within the formula.

Collectively, these four pillars of evidence provided a robust a priori safety assessment, justifying the ethical approval and subsequent investigation of SXXMKD in this clinical pilot study.

## Authentication and quality control of SXXMKD

### Authentication of raw herbal materials

All nine herbal materials were procured through the official supply chain of Zhujiang Hospital, a Grade A Tertiary Hospital in China. Our Traditional Chinese Medicine (TCM) Pharmacy, which is certified for Good Clinical Practice (GCP), handled all preparations. Upon receipt of each batch, samples were authenticated by qualified pharmacists. Conformance to the standards of the *Chinese Pharmacopoeia* (2020 edition) was confirmed via macroscopic, microscopic, and thin-layer chromatographic (TLC) analyses. Furthermore, batch-to-batch consistency was ensured using High-Performance Liquid Chromatography (HPLC) fingerprinting to guarantee the uniformity of materials throughout the study.

### Standardization of the decoction process

The decoction process was standardized to minimize variability. Preparations were made using computer-controlled, automated decoction machines following a validated Standard Operating Procedure (SOP). This SOP specifies parameters such as water volume, soaking time, decoction temperature, and duration. The entire process, from weighing the raw herbs to the packaging of the final liquid, was supervised and documented by a certified pharmacist.

### Quality control of the final product

As presented in the Supplementary Material (Supplementary Figure 1), we performed High-Performance Liquid Chromatography-Mass Spectrometry (HPLC-MS) fingerprinting on the final preparation. This served as a final chemical characterization to confirm the overall quality and batch-to-batch consistency of the decoction administered to patients, in line with the ConPhyMP recommendations.

This comprehensive quality control system, from raw material authentication to final product analysis, provided the necessary evidence to support the safe and ethical administration of SXXMKD in this prospective observational study.

## Chemical fingerprinting of the SXXMKD formula and its constituent herbs

In this study, we completed a high-resolution HPLC-MS analysis of SXXMKD, generating total ion chromatograms (TICs) in negative ion mode (see Supplementary Figure 1). Several representative peaks were annotated based on retention time and database matching. This serves as one orthogonal fingerprinting method in line with the ConPhyMP recommendations.

The herbal materials for this study’s formula-Huang Qi (15g), Tai Zi Shen (15g), Shan Zha (15g), Chuan Xiong (10g), Ge Gen (15g), Hong Jing Tian (6g), Dan Shen (15g), Mu Xiang (10g), and San Qi (10g)-were purchased from Guangdong Yuanshengtai Pharmaceutical Co., Ltd. (Guangdong, China). The nine herbal materials for the SXXMKD formula were procured through the official supply chain of Zhujiang Hospital, Southern Medical University. Upon receipt of each new batch, representative samples were drawn by the Traditional Chinese Medicine (TCM) Pharmacy of Zhujiang Hospital. These samples were then subjected to a rigorous authentication process performed by qualified harmacists. Authentication involved macroscopic, microscopic, and thin-layer chromatographic (TLC) analyses, ensuring that all materials conformed to the identity and quality standards stipulated in the Chinese Pharmacopoeia (2020 edition). Furthermore, batch-to-batch consistency was monitored using HPLC fingerprinting to guarantee the uniformity of the materials used throughout the study. Voucher specimens from each authenticated batch were cataloged, labeled with a unique identification number, and deposited at the herbarium of the TCM Pharmacy for future reference and verification.

The decoction was prepared by the Traditional Chinese Medicine (TCM) Pharmacy of Zhujiang Hospital. The raw herbs were pulverized and passed through a 60-mesh sieve. All herbs, excluding Aucklandia lappa (Mu Xiang), were soaked in 10 volumes (v/w) of purified water for 30 minutes. The mixture was brought to a boil and then simmered at (95±2)°C for 20 minutes. Subsequently, Mu Xiang (10g) was added for the final 10 minutes (late addition method). The dregs were decocted again with 8 volumes of water for 20 minutes. The filtrates from both decoctions were combined and concentrated under reduced pressure at 40°C to a final volume of 400 mL. The decocted liquid was freeze-dried to form a fine powder, which was then accurately weighed and divided into smaller portions for subsequent analysis.

The chromatographic analysis was performed at the Central Laboratory, School of Basic Medical Sciences, Southern Medical University. An accurately weighed sample of 10 mg of the lyophilized SXXMKD powder was dissolved in 1 mL of 50% methanol. The solution was vortex-mixed for 2 minutes and then centrifuged at 12,000 rpm for 10 minutes. The supernatant was filtered through a 0.22 μm syringe filter before injection into the LC-MS system.

An AB SCIEX QTRAP® 6500+ ultra-HPLC-MS/MS instrument (SCIEX, Redwood City, CA, USA) equipped with an electrospray ionization (ESI) source was used. Methanol (HPLC grade) and formic acid (HPLC grade) were purchased from Fisher Scientific (Waltham, MA, USA). The total ion chromatogram (TIC) in negative ion mode (see Supplementary Figure 1) was generated as an orthogonal fingerprinting method in line with the ConPhyMP recommendations.

Additionally, we conducted UV fingerprinting analysis for components containing UV-active compounds, such as Dan Shen and Shan Zha (see Supplementary Figure 3). However, UV spectral fingerprinting was not applicable to certain ingredients, including Huang Qi, Tai Zi Shen and San Qi, due to the absence of distinctive chromophores in the UV-visible region.

An accurately weighed sample of 1.0 g of each powdered herb (passed through a 60-mesh sieve) was placed into a 50 mL conical flask. To each flask, 50 mL of 70% ethanol was added, resulting in a drug-extract ratio (DER) of 1:50 (w/v). The mixture was then extracted using ultrasonication (40 kHz, 100W) for 30 minutes at room temperature. After extraction, the mixture was centrifuged at 3000 rpm for 10 minutes. The supernatant was collected and subsequently filtered through a 0.45 μm syringe filter into a sample vial. The resulting clear filtrate, with a concentration equivalent to 20 mg/mL, was directly used for UV-Vis spectroscopic analysis. Analyses were performed using a Shimadzu UV-1800 spectrophotometer (Kyoto, Japan), with spectra scanned from 200-800 nm at a resolution of 1 nm.

**References:**

[1] J. Ren, L. Fu, S.H. Nile, J. Zhang, and G. Kai, Salvia miltiorrhiza in Treating Cardiovascular Diseases: A Review on Its Pharmacological and Clinical Applications. Front Pharmacol 10 (2019) 753.

[2] W. Zhuang, S. Liu, X. Zhao, N. Sun, T. He, Y. Wang, B. Jia, X. Lin, Y. Chu, and S. Xi, Interaction Between Chinese Medicine and Warfarin: Clinical and Research Update. Front Pharmacol 12 (2021) 751107.

[3] C. Zhao, J. Fu, Y. Wang, and Y. Zhou, Latest Evidence and Perspectives of Panax Notoginseng Extracts and Preparations for the Treatment of Cardiovascular Diseases. J Cardiovasc Pharmacol 85 (2025) 248-260.

[4] A. Ma, F. Zou, R. Zhang, and X. Zhao, The effects and underlying mechanisms of medicine and food homologous flowers on the prevention and treatment of related diseases. J Food Biochem 46 (2022) e14430.

[5] C. Hou, X. Jiang, W. Sheng, Y. Zhang, Q. Lin, S. Hong, J. Zhao, T. Wang, and X. Ye, Xinmaikang (XMK) tablets alleviate atherosclerosis by regulating the SREBP2-mediated NLRP3/ASC/Caspase-1 signaling pathway. J Ethnopharmacol 319 (2024) 117240.

[6] M. Chen, M. Liu, X. Guo, J. Zhou, H. Yang, G. Zhong, L. Men, Y. Xie, G. Tong, Q. Liu, J. Luan, and H. Zhou, Effects of Xinkeshu tablets on coronary heart disease patients combined with anxiety and depression symptoms after percutaneous coronary intervention: A meta-analysis. Phytomedicine 104 (2022) 154243.

[7] M. Chen, G. Zhong, L. Men, Q. Liu, and J. Luan, Effectiveness and safety of Xinkeshu on coronary artery disease patients combined with anxiety and depression symptoms after percutaneous coronary intervention: A protocol for systematic review and meta-analysis. Medicine (Baltimore) 100 (2021) e27912.

[8] T.C. Wang, R.H. Zhang, P.P. Wang, R.Q. Ma, and X.F. Zhu, [Effects of xinmaikang capsule on acute myocardial ischemia in coronary artery occlusion dogs]. Zhong Yao Cai 32 (2009) 1715-9.
